# Exploiting a Neutral BODIPY Copolymer as an Effective Agent for Photodynamic Antimicrobial Inactivation

**DOI:** 10.1021/acs.jpcb.0c09634

**Published:** 2021-02-04

**Authors:** Aoibhín A. Cullen, Ashwene Rajagopal, Katharina Heintz, Andreas Heise, Robert Murphy, Igor V. Sazanovich, Gregory M. Greetham, Michael Towrie, Conor Long, Deirdre Fitzgerald-Hughes, Mary T. Pryce

**Affiliations:** † School of Chemical Sciences, Dublin City University, Dublin 9, Ireland; ‡ Department of Clinical Microbiology, RCSI Education and Research, Royal College of Surgeons in Ireland, Beaumont Hospital, Beaumont, Dublin 9, Ireland; § Department of Chemistry, Science Foundation Ireland (SFI) Centre for Research in Medical Devices (CURAM), The Science Foundation Ireland (SFI) Advanced Materials and Bioengineering Research Centre (AMBER), RCSI University of Medicine and Health Science, 123 St. Stephen’s Green, Dublin 2, Ireland; ∥ Central Laser Facility, Science & Technology Facilities Council, Research Complex at Harwell, Rutherford Appleton Laboratory, Didcot OX11 0QX, U.K.

## Abstract

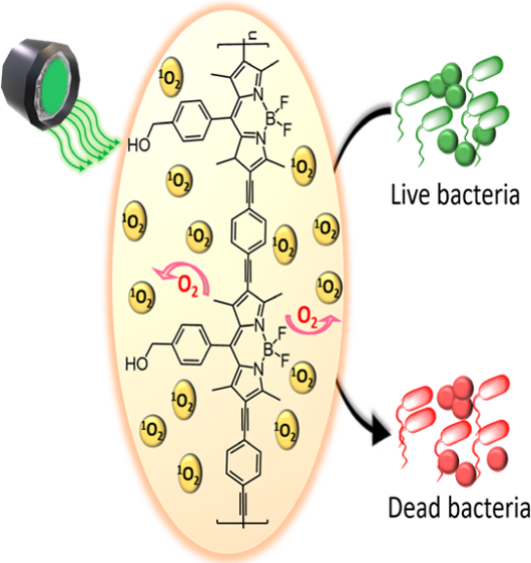

We report the synthesis and photophysical properties of a neutral BODIPY photosensitizing copolymer (poly-8-(4-hydroxymethylphenyl)-4,4-difluoro-2,6-diethynyl-4-bora-3a,4a-diaza-*s*-indacene) containing ethynylbenzene links between the BODIPY units. The copolymer absorbs further towards the red in the UV-vis spectrum compared to the BODIPY precursor. Photolysis of the polymer produces a singlet excited state which crosses to the triplet surface in less than 300 ps. This triplet state was used to form singlet oxygen with a quantum yield of 0.34. The steps leading to population of the triplet state were studied using time-resolved spectroscopic techniques spanning the pico- to nanosecond timescales. The ability of the BODIPY polymer to generate a biocidal species for bactericidal activity in both solution- and coating-based studies was assessed. When the BODIPY copolymer was dropcast onto a surface, 4 log and 6 log reductions in colony forming units/ml representative of Gram-positive and Gram-negative bacteria, respectively, under illumination at 525 nm were observed. The potent broad-spectrum antimicrobial activity of a neutral metal-free copolymer when exposed to visible light conditions may have potential clinical applications in infection management.

## Introduction

The increasing resistance of bacterial pathogens to antibiotics is a growing problem, particularly in healthcare settings.^1,2^ Conventional antibiotics target the cell wall, DNA, or protein synthesis, all of which are critical to bacterial survival. Unfortunately, infections such as those caused by methicillin-resistant *Staphylococcus aureus* or β-lactamase-producing Enterobacteriales are increasing and the effectiveness of many antibiotics in clinical use is declining.^3^ Alternative multimodal approaches are now required to manage these life-changing or life-threatening infections.^4^

Photodynamic therapies have been used in various cancer treatments.^5−9^ In contrast, antimicrobial photodynamic therapies (aPDTs) are less well developed, although interest in this approach is increasing. These therapies rely on a mechanism that involves the production of reactive oxygen species (ROS), mainly singlet oxygen, which disrupts the bacterial cell membrane and other critical cellular processes.^10^ In the aPDT approach, photosensitizers are required to absorb photon energies in an appropriate spectral region. Photosensitizers (PSs), with extensive π-conjugation such as porphyrins, (tetrapyrrole macrocycles resembling biological organic components),^11,12^ chlorins, and phthalocyanines,^13−15^ have been used in aPDTs, and several of these have been approved for clinical use or are currently in clinical trials.^16^ The excellent photophysical properties such as high molar extinction coefficient in the visible region, long emission wavelength, high fluorescence quantum yield, photostability, and low dark toxicity have ensured an increased interest in the use of boron dipyrromethenes (BODIPYs) as photosensitizers. In addition, the chemical versatility of BODIPYs enables a fine tuning of the photophysical processes by changing the nature and location of substituents on the BODIPY core. The BODIPY derivatives have found applications in therapeutics, theragnostics, drug delivery agents, fluorescent switches, and other industrial applications.^17−21^

Singlet oxygen is generated by energy transfer from a triplet state photosensitizer (^3^PS).^22,23^ However, excitation of BODIPY compounds produces singlet excited states which emit with high fluorescence quantum yields (Φ_fl_).^17,24^ Nonetheless, the synthetic versatility of the BODIPY core provides several approaches to enhance the triplet state formation. One of these is to introduce a heavy element, such as iodine, which promotes greater spin–orbit coupling and crossing to the triplet manifold following excitation. While this synthetic modification enhances antimicrobial activity, it also results in high levels of dark toxicity, which is undesirable in therapeutic applications.^25−31^

There are a few reports of heavy atom-free BODIPY systems which can generate triplet excited states.^19,32−38^ The structural modifications to the BODIPY core required to promote triplet formation have been reviewed recently.^21,39−42^ Orlandi et al. reported that introducing cationic pyridinium groups to the *meso* position in the BODIPY core can enhance their performance in aPDTs.^43^

An alternative approach is to add an acceptor or donor site to the BODIPY core. The excited states involving charge transfer to/from the BODIPY core are then accessible.^44^ The addition of extended conjugation allows the charge separation in these excited states to be increased, and this promotes crossing to the triplet surface.^45^ Extended conjugation of this type also results in a red shift of the absorption bands toward the “therapeutic window”.^46,47^ Li et al. showed that cationic polymers based on copolymerization of BODIPY with 2-(dimethylamino)ethylmethacrylate and galactose produce extensive PDT activity and increased cytocompatibility in dark conditions.^48−50^ Cationic BODIPY compounds have exhibited improved aPDT performance when polyamidoamines are used as adjuvants. Increased penetration of the lipid-rich layers of the outer membrane/cytoplasmic membrane was observed under these conditions. Copolymers of the BODIPY core with π-conjugated comonomers have lower band gaps and better optical properties including broader absorption profiles.^47,51^

Building on these studies, we have synthesized a π-conjugated BODIPY copolymer with 1,4-diethynylbenzene. The BODIPY core contains a phenyl alcohol at the *meso* position. The photophysical properties of the copolymer were investigated using both picosecond (ps) and nanosecond (ns) transient absorption and time-resolved infrared spectroscopy. The polymer was assessed for antibacterial activity under visible light irradiation, with a resulting log 4–6 reduction against Gram-positive and Gram-negative bacteria, including multidrug-resistant strains. To our knowledge, this is the first example of a copolymer that does not contain a metal or heavy atom that demonstrates potent antimicrobial properties in combination with visible light.

## Results and Discussion

**Figure 1:**
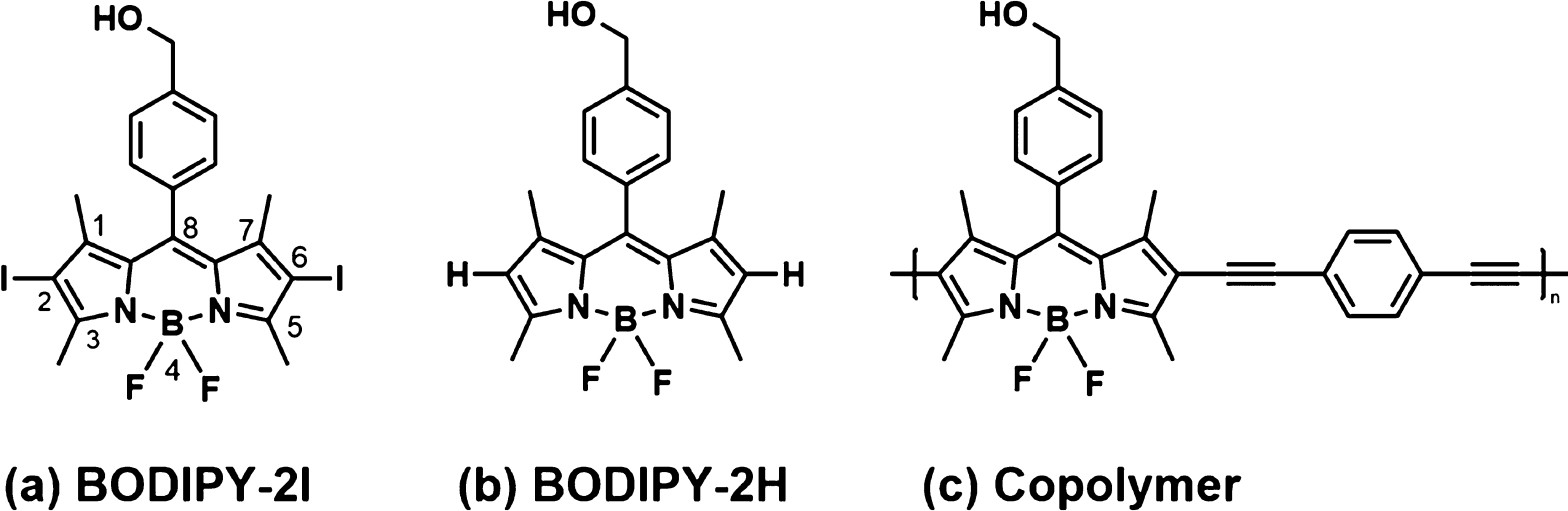
(a) Chemical structure of 2,6-diiodo-8-(4-hydroxymethylphenyl)-4,4′difluoro-1,3,5,7-tetramethyl-4-bora-3a,4a-diaza-*s*-indacene (BODIPY-2I), (b) 8-(4-hydroxymethylphenyl)-4,4′difluoro-1,3,5,7-tetramethyl-4-bora-3a,4a-diaza-*s*-indacene (BODIPY-2H), and (c) the repeat unit of the copolymer.

The copolymer was synthesized using the Sonogashira coupling between the diiodo BODIPY (Figure 1a) and diethynylbenzene comonomers (Supporting Information, section 2). Size exclusion chromatography (SEC) revealed a monomodal weight distribution with some high molecular weight tailing (Supporting Information, Figure S4). While exact molar masses cannot be determined because of the structural discrepancy between the calibration standard PMMA and the copolymer structure, SEC analysis confirms the successful formation of the copolymeric material. However, the presence of some low molecular weight oligomeric structures is also likely. Figure 1b represents the model BODIPY-2H which was used as a model for the photosensitizer and Figure 1c represents the repeat unit of the copolymer. (See Supporting Information sections 2, 3).

**Figure 2:**
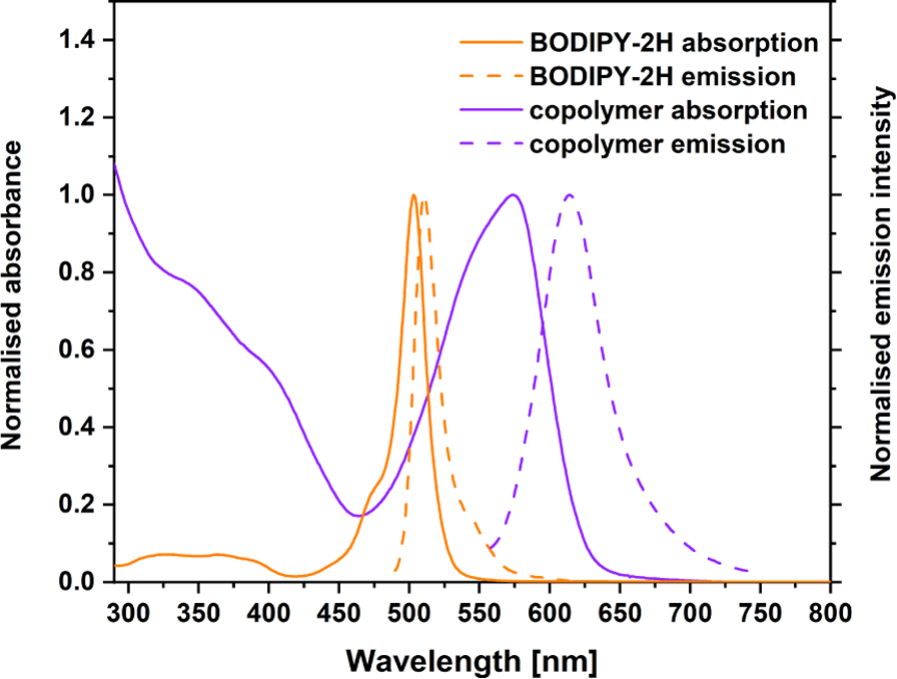
Normalized UV–visible absorption spectra of BODIPY-2H (orange solid line) and the copolymer (purple solid line), and normalized emission spectra of BODIPY-2H (orange dashed line) (λ_exc_ = 503 nm) and the copolymer (purple dashed line) (λ_exc_ = 560 nm). The e All spectra were recorded in CH_2_Cl_2_ at room temperature.

The UV–visible spectrum for the BODIPY-2H (λ_max_ = 503 nm) is typical for BODIPY compounds. The lowest energy absorption populates the lowest energy singlet excited state. In comparison, the copolymer exhibits a λ_max_ at 574 nm and the absorption band is broader (Figure 2). This can be explained by enhanced conjugation along the backbone of the copolymer.^52,53^ The photophysical properties of BODIPY-2H were measured in a variety of solvents (see Supporting Information sections 4, 5, 6) and are typical of BODIPY compounds. The fluorescence quantum yield (Φ_fl_) of 0.75 is typical of BODIPY compounds, where fluorescence efficiency close to unity is widely reported with emission lifetimes of 5 ns or less.^54−56^ The emission lifetime of BODIPY-2H was measured at 5.80 ns ( ±0.09 ns), and this is consistent with previous reports. In the case of the copolymer, a red shift in the emission maxima was also observed when compared to BODIPY-2H, together with a large Stokes shift of the emission (1128 cm^–1^ compared to 273 cm^–1^ for BODIPY-2H) (Figure 2). In addition, the luminescence quantum yield for the copolymer was considerably lower at 0.17 than for BODIPY-2H. This difference suggests the presence of a radiationless pathway to deactivate the lowest energy singlet excited state in the copolymer, which is not available to BODIPY-2H. This involves a transition to the triplet surface possibly by way of a singlet fission process.^57−62^ The emission lifetime measurements for the copolymer suggest that the emission follows a biexponential decay profile yielding two lifetimes [τ_fl_ = 0.90 ns ( ±0.03 ns) and 2.89 ns ( ±0.05 ns)]. One explanation for these emissions is that they originate from spin-correlated triplet pairs produced in the singlet fission process. Such triplet pairs are known to emit weakly.^63−66^ The observation of two emissive species suggests the presence of two emissive conformations on the copolymer backbone, with one more localized near the BODIPY units and the other delocalized along the copolymer backbone.

Following excitation of the copolymer at 525 nm, the characteristic emission band of singlet oxygen (∼1270 nm) was observed, with a quantum yield (Φ_Δ_) of 0.34 (see Table 1, Supporting Information section 6, Figure S8), using rose bengal as the standard.^22,67,68^ This observation further confirms that a triplet state must be populated in this copolymer. In contrast, similar excitation of BODIPY-2H produced no detectable singlet oxygen, which is in agreement with the literature.

**Table 1:** The photophysical properties of BODIPY-2H and the Copolymer were determined in CH_2_Cl_2_

compound	λ_abs_ (nm)	λ_em_ (nm)	Φ_fl_^c^	τ^d^	Φ_Δ_^e^
BODIPY-2H	503	510^a^	0.75	5.80 ± 0.09	
copolymer	574	614^b^	0.17	0.90 ± 0.03, 2.89 ± 0.05	0.34

a λ_exc_ = 490 nm.

b λ_exc_ = 560 nm.

c 3-pyridine H-BODIPY used as standard: Φ_fl_ = 0.62 in CH_2_Cl_2_.^69^

d λ_exc_ = 510 nm.

e Rose Bengal used as standard: Φ_Δ_ = 0.53 in CH_3_CN.^70^ For full experimental details, see Supporting Information.

### Picosecond Transient Absorption

Excitation of the copolymer at 525 nm resulted in a ground state bleach (GSB) for all solvent systems investigated, with concomitant formation of an excited state absorption (ESA) at approximately 450 nm. On this timescale, the GSB overlaps with the stimulated emission (SE), which occurs in the region 500–750 nm for the copolymer. Notably, changing the solvent polarity does not significantly affect the transient absorption spectra. Global analysis of the transient data matrix revealed three components (Supporting Information, Figure S10 and Table 2). The first component which decays over 4–20 ps (depending on the solvent) is assigned to a very rapid structural change to the copolymer.^71−73^ The second component, (τ_2_), has lifetimes in the range 220–280 ps depending on the solvent and is assigned to the singlet excited state, which decays to produce the triplet state. If population of the triplet state occurs via a singlet fission mechanism, the persistent triplet absorption may initially involve spin-correlated triplet pairs. Alternatively, as described in recent studies, twisted intramolecular charge-transfer states may be involved as precursors to the triplet state.^74,75^

**Table 2:** Summary of Lifetimes Obtained Using Global Analysis from psTAS Experiments in Different Solvents Following Excitation at 525 nm^a^

solvent	*E*_T_(30)	λ_max_ (of ESA) (nm)	τ_1_ (ps)	τ_2_ (ps)
CD_3_CN	45.6	464	4 ± 0.1	251 ± 6
DMSO-*d*_6_	45.1	461	14 ± 0.3	219 ± 5
CD_2_Cl_2_	40.7	459	26 ± 0.6	287 ± 5

a The Reichardt parameter *E*_T_(30) is shown to indicate solvent polarity.^77^

BODIPY-2I reveals significant differences in the excited-state spectra of both the copolymer and BODIPY-2H. Initially, an ESA feature is apparent at 472 nm corresponding to the singlet excited state (S_1_), which undergoes intersystem crossing (ISC) to the triplet state (τ of S_1_*ca*.146 ps) (Figure S9). Transient absorption studies of other halogenated BODIPY compounds report similar spectroscopic features which have been assigned to triplet excited states.^20,76^ While the transient absorption studies indicate that both the copolymer and BODIPY-2I form a triplet excited state, the time-resolved experiments indicate that in the case of the copolymer, the photophysics is much more complex. While the precise mechanism for the formation of triplet excited states within the copolymer remains uncertain, a number of possible mechanisms exist including the formation of charge-transfer excited states which is known for many BODIPY compounds, but another possibility includes singlet fission.

**Figure 3:**
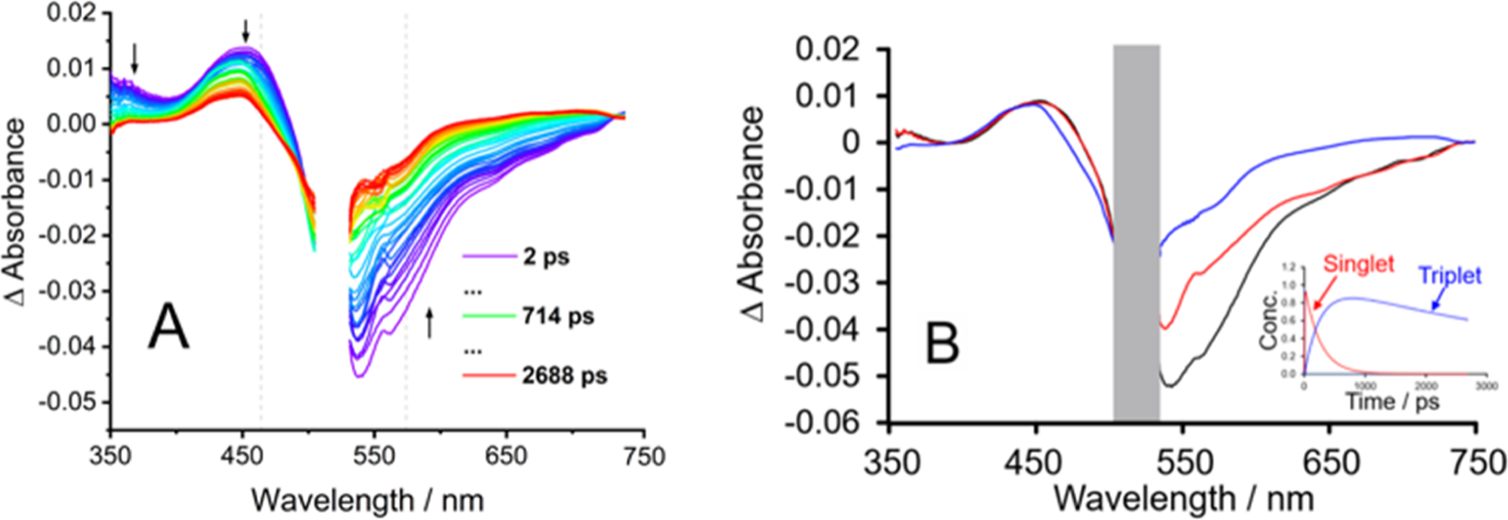
Transient absorption (TA) spectra of (A) the copolymer in acetonitrile and (B) Evolution associated spectra (EAS) in acetonitrile. λ_exc_ = 525 nm (0.4 μJ/pulse) (the gap in the TA plots corresponds to the excitation wavelength which is masked by a narrow band pass filter).

The photophysics of BODIPY-2H is less complex than those of either BODIPY-2I or the copolymer. Following excitation of BODIPY-2H, a GSB occurs, together with an ESA feature that is produced at 430 nm (Figure S11) corresponding to the lowest energy singlet excited state (S_1_). This absorption decays on a timescale similar to the emission lifetime (5.8 ns, Table 1), although it is not possible to monitor the full decay because the lifetime lies outside the time response window of the transient absorption apparatus (3 ns) (Figure 3).

### Nanosecond Transient Absorption

**Figure 4:**
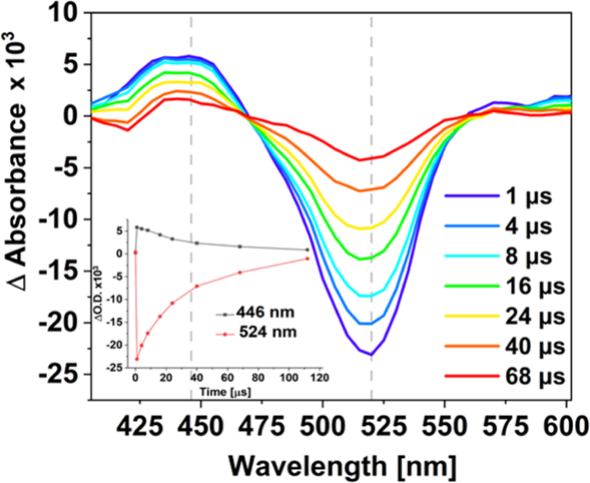
TA spectra of the copolymer in CH_3_CN at different time delays (<1–68 μs). The sample was degassed using three freeze–pump–thaw cycles. The gray dashed line on TA spectra indicates the wavelengths chosen for the kinetic traces shown in the inset, which were used to determine the decay lifetimes.

Nanosecond transient absorption spectra were measured in acetonitrile, following freeze–pump–thawing of the samples and excitation at 355 nm using a LP980 transient absorption spectrometer (Edinburgh instruments as described in the Supporting Information). These studies confirmed the formation of a long-lived excited state. The spectral features obtained in these experiments are similar to the persistent species observed in the picosecond transient absorption (psTA) experiments (λ_exc_ = 525 nm). An ESA is evident in the range 420–470 nm, together with a bleach in the range of 500–550 nm, similar to that in the psTA spectroscopic studies. The recovery of the parent bleach occurs with the same rate as the band at 440 nm decays (inset in Figure 4). These spectra are consistent with the decay of a triplet species with τ_T_ = 32 μs (Figure 4).

### Time-Resolved Infrared Spectroscopy

**Figure 5:**
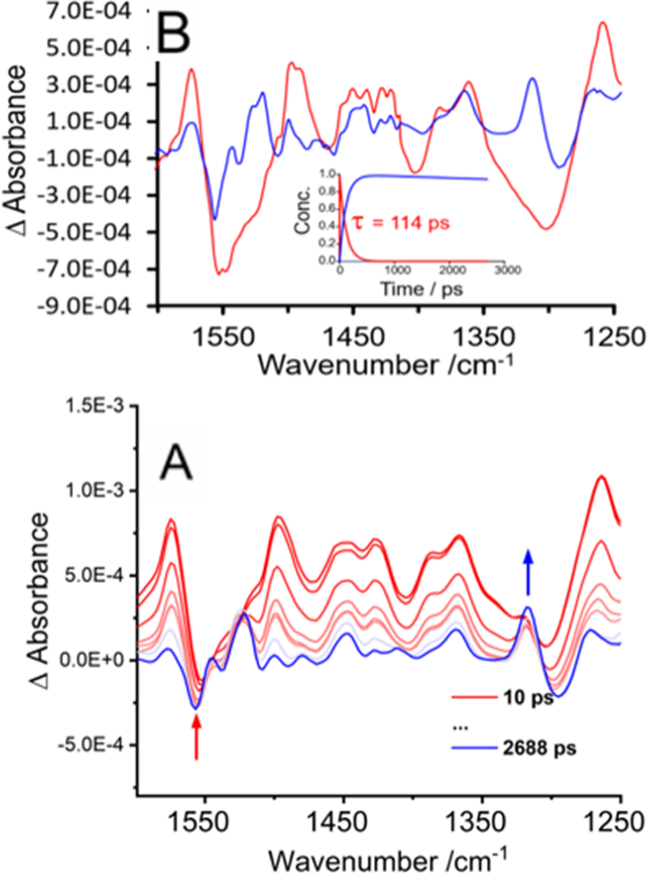
(A)TRIR spectra of the copolymer in CD_3_CN in the spectral window of 1610–1250 cm^–1^ at various time delays following excitation at 525 nm. (B) and the corresponding EAS.

Displayed in Figure 5 are the time-resolved infrared spectra (TRIR) of the copolymer following excitation at 525 nm in CD_3_CN. Following excitation, bleaching of the parent (attributed to the BODIPY core mixed with the phenyl modes) is evident at 1552, 1469, and 1308 cm^–1^ together with new features at 1574, 1498, 1452, 1366, and 1261 cm^–1^. Further features appear at 1552, 1469, and 1308 cm^–1^.^78^ Global analysis of the data matrix revealed using a sequential model of two components, the first is assigned to the S_1_ species which decays with a lifetime of 114 + 5 ps forming a second persistent component assigned to the triplet excited state. The spectra for these two species are displayed in Figure 5. These results are similar to those obtained in the psTA experiments described above. The TRIR experiments were also carried out in CD_2_Cl_2_ and DMSO and confirmed that the spectral features were not sensitive to the solvent medium (Supporting Information section 9, Figures S13 and S14, respectively).

The TRIR spectra were also obtained for the corresponding BODIPY-2H and BODIPY-2I compounds. In the case of BODIPY-2I, following excitation, depletion of the parent is evident at 1537 cm^–1^ and a band at 1490 cm^–1^ assigned to the singlet species is formed. This latter band decays over 80 ps, giving rise to a further band at 1523 cm^–1^ assigned to the triplet excited state (that persists on the nanosecond timescale).^76^ In the case of BODIPY-2H, photolysis results in the depletion of the parent bands, and new bands at 1551, 1512, 1451, 1416, and 1276 cm^–1^ are formed. Analysis of the data indicates two components, a short-lived species that decays within 20 ps. As there is no significant shift in the infrared stretching vibrations, these initial IR bands are assigned to structural relaxation from the Franck–Condon state. A further species, assigned to the singlet state (S_1_) persists onto the nanosecond time scale (Supporting Information, Figure S15 and Figure S16).

**Figure 6:**
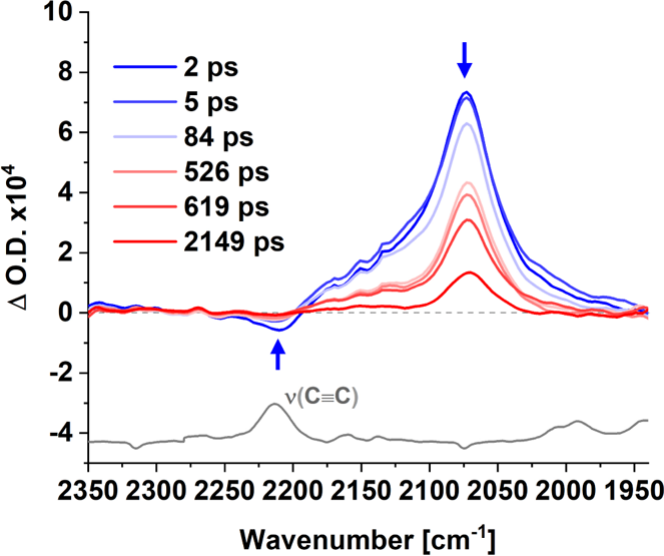
TRIR spectra of the copolymer in CHCl_3_ following pulsed photolysis (λ_exc_ = 525 nm) in the triple bond region recorded at various time delays, with arrows indicating the time-dependent behavior of the spectral features. FTIR of copolymer displayed in the same IR region (gray solid line) for reference.

TRIR spectra in the carbon–carbon triple bond region were also obtained. Following excitation (525 nm), the weak parent band at 2203 cm^–1^ was depleted and a more intense feature at 2073 cm^–1^ was produced (Figure 6). This shift to lower energy indicates a decreased electron density on the π linker along the copolymer backbone^78^ (confirmed from FTIR, Supporting Information section 8, Figure S12). The intensity of the feature at 2073 cm^–1^ relative to the ground-state bleach is large and can be explained by a considerable change in the dipole moment in these excited states.^79^ This is strong evidence to support a charge-transfer character of both singlet and triplet excited state species. This feature decays with concomitant recovery of the parent bleach as indicated by the blue arrows in Figure 6. Kinetic analysis of this signal yielded lifetimes of τ_1_ = 4.9 ± 5 ps and τ_2_ = 131 ± 20 ps in CHCl_3_ (Figure S17). A further component that persists well into the ns timescale (≫ 3 ns) is consistent with the formation of a long-lived triplet species. The short-lived component (τ_1_) is assigned to a structural change along the polymer backbone, with τ_2_, corresponding to the decay of singlet to form the triplet excited state.

### Antimicrobial Photodynamic Inactivation

Singlet oxygen generation (^1^O_2_) has been reported to contribute marked photoactive bactericidal properties to different chromophores.^80,81^ In the present study, for antimicrobial evaluation, a solution-based bactericidal assay was performed on laboratory strains of *S. aureus* (ATCC 25923), methicillin-resistant *S. aureus* [(MRSA), (ATCC 43300)], *Escherichia coli* (ATCC 25922), and an extended spectrum β-lactamase (ESBL) producing *E. coli* clinical isolate (CL11). Experimental details of assay preparation are provided in the Supporting Information. Solvatochromic studies using UV–visible spectroscopy confirmed the stability of the copolymer in DMSO/PBS (PBS = phosphate buffered saline) mixture relevant to the assay conditions (see Supporting Information section 10, Figure S18). The assays were irradiated under light of wavelength, λ = 525 nm. Control assays containing no copolymer (growth control) and nonirradiated samples (dark control) were included.

**Figure 7:**
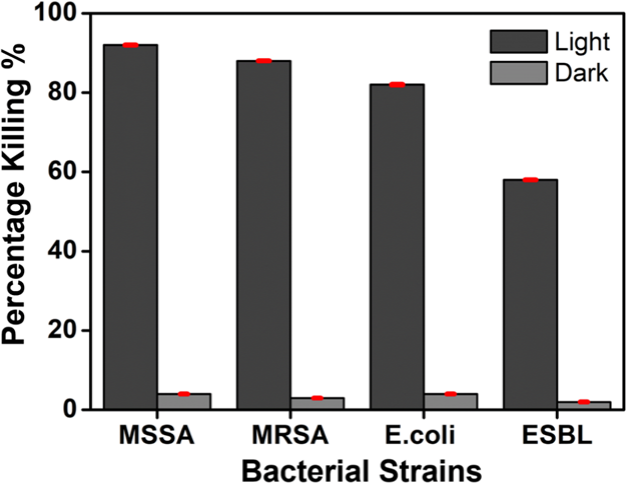
Antimicrobial activity of the copolymer under irradiation and nonirradiation conditions, with *S. aureus* [ATCC 25923 and ATCC 43300 (MRSA)] and *E. coli* [ATCC 25922 and CL11 (ESBL)], [copolymer] for *S. aureus*: 1 μg/mL, [copolymer] for *E. coli*: 5 μg/mL, time of irradiation: 15 min, wavelength of light for irradiation λ ∼ 525 nm. The data represent percentage killing (CFU/mL relative to controls) and are the mean ± SEM of three assays carried out in triplicates.

Antibacterial susceptibility testing indicated greater activity of the copolymer compared to the monomer at 1 and 5 μg/mL for Gram-positive and Gram-negative bacteria, respectively (Supporting Information section 10, Figure S19). In darkness, the complexes had a negligible effect on bacterial growth. Light irradiation at 525 nm for ∼15 min and concentrations of 1 and 5 μg/mL of the copolymer resulted in a photoactivated bactericidal effect (Figure 7). At 1 μg/mL, the copolymer led to a >80% killing of MRSA and methicillin-susceptible *S. aureus* (MSSA). Killing of Gram-negatives (*E. coli* and ESBL *E. coli*) was not as effective, even at a higher concentration of 5 μg/mL. In contrast, the monomer exhibited negligible antibacterial activity in the same conditions.

Concentration dependence of bactericidal activity was observed for the copolymer in the range 1–5 μg/mL. However, the formation of interconnected aggregates at concentrations of copolymer >10 μg/mL precluded accurate determination of antimicrobial activity in solution beyond this concentration. Therefore, the copolymer was drop-coated onto surfaces for increased stability and to facilitate investigation of antimicrobial activity at higher concentrations.

**Figure 8:**
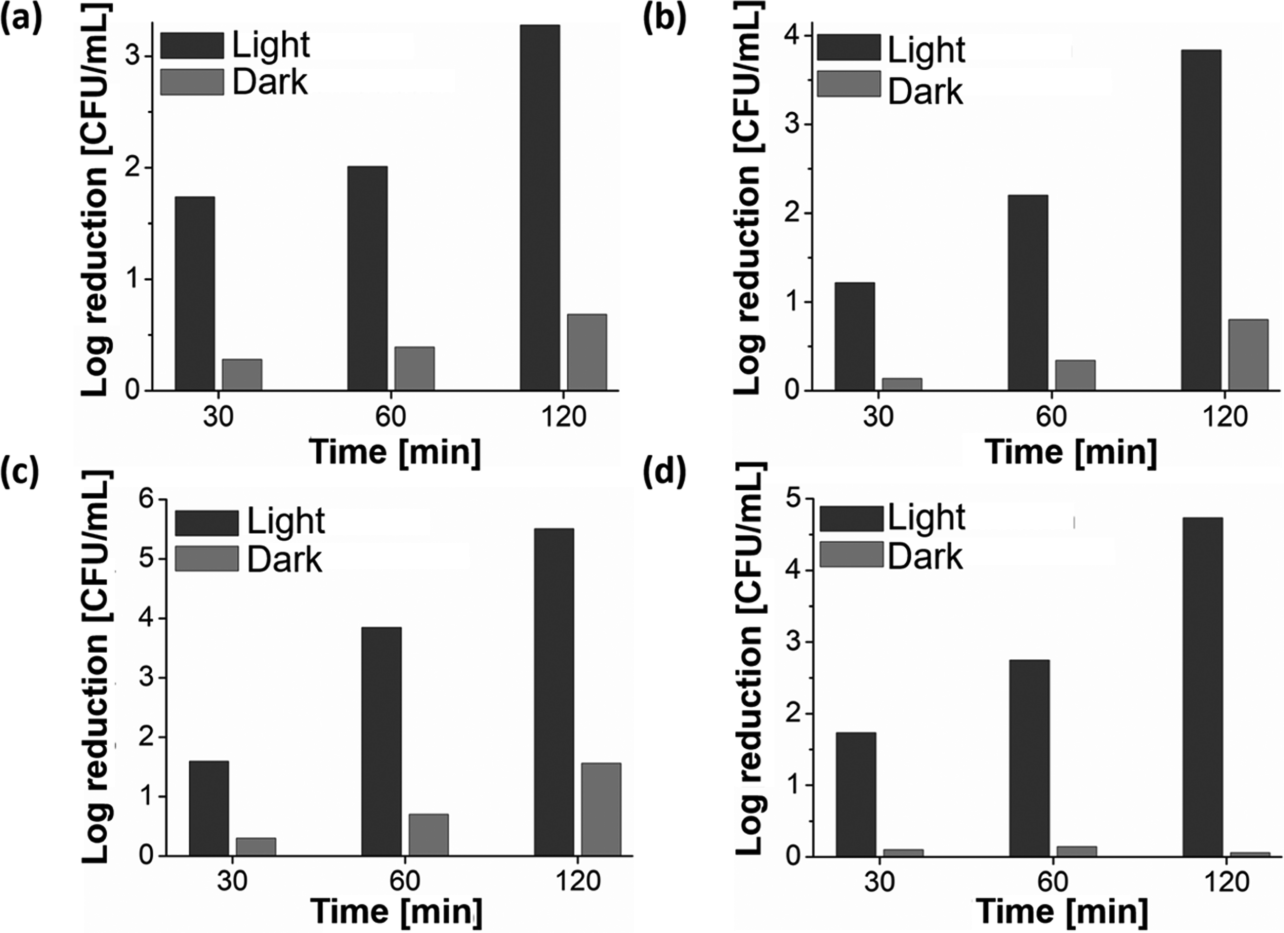
Comparison of the antimicrobial activity of the drop-coated copolymer on 96-well plates under irradiation and nonirradiation conditions for (a) *S. aureus* (ATCC 25923), (b) MRSA (ATCC 43300), (c) *E. coli* (ATCC 25922), and (d) ESBL (CL11), [copolymer]: 40 μg/mL, varied irradiation time, wavelength of light for irradiation λ ∼ 525 nm.

Experimentally, the wells of a 96-well plate were drop-coated with the copolymer (40 μg/mL) which was air-dried in a laminar flow cabinet before addition of the bacterial suspension. Using this method, a homogeneous surface of the copolymer coating was observed with SEM measurement performed before irradiation (Supporting Information section 3.4, Figure S5). No steric hindrance-associated aggregate formation was observed for the copolymer.^82^ This indicates a more stable and an improved ordering of the BODIPY core and planarity of the copolymer backbone. The surface was irradiated using visible light (λ = 525 nm). Greater bactericidal activity of the copolymer was observed for all bacteria when tested under coating conditions and for longer light exposure times, compared to aqueous conditions. Killing efficacy reached 5–6 log reduction for *E. coli* and 3–4 log reduction for *S. aureus*. Similar killing efficacy was observed for the multidrug-resistant strains compared to the antibiotic susceptible strains of each genera (Figure 8). The greater relative killing of *E. coli* compared to *S. aureus* when drop-coated was unexpected as studies of other BODIPYs, albeit in solution, have shown lower activity toward Gram-negatives, based on their additional outer membrane.^83^

Measurement of the copolymer cytotoxicity to cultured human keratinocytes (HaCaT cells) using the MTT assay revealed an IC_50_ value of 45.2 μg/mL (Supporting Information section 11, Figure S20). This is the concentration resulting in the loss of viability of up to 50% of HaCaT cells. This indicates some cytotoxicity to keratinocytes at concentrations close to those that resulted in >5 log reduction in Gram-negative and >3 log reduction to Gram-positive bacteria.

## Conclusions

In this work, we report a straightforward synthesis of a metal and heavy atom-free neutral BODIPY-containing copolymer which has potent biocidal properties when irradiated with visible light (λ > 500 nm). A detailed investigation into the photophysical properties was performed using ps–ns transient absorption and time-resolved infrared spectroscopic techniques on both the copolymer and the corresponding BODIPY photosensitizers. These studies provided insights into the photophysical properties of both the copolymer and BODIPY-based photosensitizers. In the case of the copolymer, we have tentatively assigned the pathway to singlet oxygen generation to occur via a singlet fission mechanism. Our research is currently focused on obtaining more definite evidence for this. Under irradiation with visible light, the copolymer produces singlet oxygen which explains the antimicrobial activity of this neutral polymer toward Gram-negative bacteria. This work provides us with a basis where singlet fission can be exploited for the development of more potent polymeric-based materials with low cytotoxicity as surface coatings for antimicrobial therapeutics.

## Supporting Information Available

Details on the experimental methods, synthesis procedure, instrumentation, and different characterization methods used to perform this study (PDF)
