# Supplementary material for: Exploiting a Neutral BODIPY Copolymer as an Effective Agent for Photodynamic Antimicrobial Inactivation
Source: J Phys Chem B. 2021 Feb 4;125(6):1550–7. doi: 10.1021/acs.jpcb.0c09634 (PMC8279490; doi:10.1021/acs.jpcb.0c09634)
Supplement: Supplementary file 1 — jp0c09634_si_001.pdf [file jp0c09634_si_001.pdf]

Electronic Supplementary Information

Exploiting a Neutral BODIPY Copolymer as an Effective Agent for Photodynamic Antimicrobial Inactivation

Aoibhin A. Cullen[a], Ashwene Rajagopal[a,b], Katharina Heintz[a], Andreas Heise[c], Robert Murphy[c], Igor V. Sazanovich[d], Gregory M. Greetham[d], Michael Towrie[d], Conor Long[a], Deirdre Fitzgerald-Hughes[b]*, Mary T. Pryce[a]*

[a] School of Chemical Sciences, Dublin City University, Dublin 9, Ireland.

[b] Department of Clinical Microbiology, Royal College of Surgeons in Ireland, RCSI Education and Research, Beaumont Hospital, Beaumont, Dublin 9, Ireland.

[c.] Department of Chemistry, RCSI University of Medicine and Health Science, 123 St. Stephen's Green, Dublin 2, Ireland, Science Foundation Ireland (SFI) Centre for Research in Medical Devices (CURAM),

The Science Foundation Ireland (SFI) Advanced Materials and Bioengineering Research Centre (AMBER),

[d] Central Laser Facility, Science & Technology Facilities Council, Research Complex at Harwell, Rutherford Appleton Laboratory, Didcot, OX11 0QX, UK.

Table of Contents

1. Experimental section **5**

1.1 Instrumentation **5**

1.2 Experimental Methods **6**

2 Synthesis **8**

3 Characterisation **11**

3.1 NMR spectra **11**

3.2 Mass spectroscopy **12**

3.3 Size Exclusion Chromatography **13**

3.4 Stability of drop coated polymer surface **14**

4 Absorption and emission spectra **15**

5 Lifetime measurements (TSCPC measurements) **16**

6 Singlet oxygen measurements **17**

7 Transient absorption (TA) spectroscopy **18**

7.1 Picosecond Transient Absorption 18

8 FTIR spectra 21

9 Time resolved Infrared Spectroscopy (TRIR) 22

10 Antimicrobial Evaluation 25

11 Cytotoxic study using MTT assay 27

12 References 29

Table of Figures

Figure S1. ^{1}H NMR (600 MHz) of BODIPY-2H in $CDCl_3$. 11

Figure S2. ^{1}H NMR (600 MHz) of BODIPY-2I in $CDCl_3$........ 11

Figure S3. Mass spectra for [M+] for BODIPY-2I, diiodo monomer. 12

Figure S4. Size Exclusion chromatography (SEC) trace of copolymer (dRI detection, PMMA standards). 13

Figure S5. a) UV-visible spectra of OH copolymer drop coated onto a cuvette surface. b) SEM image representing the morphology of the OH copolymer when drop coated on a surface. 14

Figure S6. a) UV-visible absorption spectra of copolymer in a range of solvents; dichloromethane (solid magenta), DMSO (solid blue), THF (solid red), toluene (solid cyan), acetone (solid violet), acetonitrile (solid black), chloroform (solid green) and b) Emission spectra of copolymer in same range of solvents, λ_{exc} = 560 nm, 15 nm slit width. Inset shows normalised emission spectra of copolymer. All spectra recorded at room temperature in aerated solution. 15

Figure S7. Emission decay profiles of a) BODIPY-2H in CH_2Cl_2, b) copolymer in CH_2Cl_2, c) copolymer in CH_3CN and d) copolymer in DMSO. The λ_{exc} = 510 nm for all samples, and a detection wavelength of 520 nm for BODIPY-2H and 610 for the copolymer. All emission decays recorded at room temperature in aerated solution. 16

Figure S8. NIR singlet oxygen emission spectrum of Rose Bengal (red), copolymer (purple), BODIPY-2H (orange) in CH_3CN, following excitation at 525 nm. 17

Figure S9. a) TA spectra of diiodo monomer (BODIPY-2I) in CH_3CN shown at indicated time decays in ps and b) corresponding kinetic traces at 430 nm (blue) and 473 nm (red). The red line corresponding to the kinetic trace at 473 nm shows the monoexponential fit used to obtain S_1 lifetime of halogenated species. As the signal at 473nm decays (τ = 146 ps), an additional species grows in at 430 nm (τ = 137 ps). These are assigned to the decay of the BODIPY singlet state and formation of the triplet state which persists beyond the time frame of the experimental set-up. The grey dashed lines on the TA spectra correspond to kinetic traces on the right. The asterisk indicates the isosbestic point at 463 nm. λ_{exc} = 525 nm (0.4 μJ/pulse). 18

Figure S10. a) TA spectra of copolymer in CH_2Cl_2 at various time delays indicated in ps with b) corresponding kinetic traces for ESA feature displayed a 440 nm (green circles), 460 nm (blue circles) and 465 nm (red circles). Fit lines for biexponential function are shown in solid lines with corresponding colour. Grey dashed line on TA spectra indicate the kinetic traces on the right. λ_{exc} = 525 nm. 19

Figure S11. a) TA spectra of H-monomer (BODIPY-2H) in CH_3CN shown at indicated time decays in ps and b) corresponding kinetic traces at 431 nm (red) and 545 nm (grey). The grey dashed lines on the

TA spectra correspond to kinetic traces on the right which do not decay to the baseline within the timeframe of the experiment. λ_{exc} = 525 nm (0.4 μJ/pulse). 20

Figure S12. FTIR spectra of 1,4 diethynylbenzene (black), BODIPY-2H (blue), BODIPY-2I (red) and copolymer (green) recorded in dichloromethane solution. Inset displays triple bond region 2400 – 2000 cm^{-1}.......... 21

Figure S13. TRIR spectra of copolymer in CD_3CN in the spectral window of 1610 – 1290 cm^{-1} at various time delays following excitation at 525 nm. Arrows display spectral changes observed throughout the course of the experiment. 22

Figure S14. TRIR spectra of copolymer in DMSO in the spectral window of 1610 – 1290 cm^{-1} at various time delays following excitation at 525 nm. Arrows display spectral changes observed throughout the course of the experiment. 22

Figure S15. TRIR spectra of BODIPY-2I in CD_3CN in the spectral window of 1610 – 1290 cm^{-1} at various time delays following excitation at 525 nm. Arrows display spectral changes observed throughout the course of the experiment.......... 23

Figure S16. TRIR spectra of BODIPY-2H in CD_3CN in the spectral window of 1610 – 1290 cm^{-1} at various time delays following excitation at 525 nm. Arrows display spectral changes observed throughout the course of the experiment. 23

Figure S17. TRIR spectra the copolymer in $CHCl_3$ following pulsed photolysis (λ_{exc} = 525 nm) in the triple bond spectral region, recorded at various time delays, with arrows indicating the time dependent behaviour of the spectral features. Inset displays the kinetic traces at both 2103cm^{-1} and 2073cm^{-1} (corresponding to both red and blue dashed lines on spectra) and the biexponential fit (solid red and solid blue line) to obtain the decay lifetimes. 24

Figure S18. UV-visible absorption spectra of copolymer in a range of solvents; especially depicting the stability of the compound in a DMSO/PBS solvent mixture (cyan colour). 25

Figure S19. Comparison on the photoactivated bactericidal activity of copolymer and its BODIPY-2H, Conditions: Bacterial strains Staphylococcus aureus (MSSA, ATCC 25923), methicillin-resistant S.aureus (MRSA, ATCC 43300), Esherichia.coli (E.coli, ATCC 25922) and an extended spectrum β-lactamase (ESBL) producing E.coli (CL11), [copolymer/BODIPY-2H] for gram-positive bacteria: 1 µg/ml, [copolymer/monomer] for gram-negative bacteria: 5µg/ml, time of irradiation: 15 minutes, wavelength of light for irradiation λ ~525 nm. 26

Figure S20. The half maximal inhibitory concentration (IC_{50}) curve of OH copolymer under varied concentration on the keratinocyte HaCaT cells. 28

1. Experimental section

1.1 Instrumentation

Steady-state spectroscopy: UV-vis spectra was measured using an Agilent 8453 UV-vis spectrophotometer in a 1 cm quartz cell using spectroscopic grade solvents. Emission spectra were recorded using the FLS1000 Edinburgh Instruments equipped with a Xe Arc lamp and a visible PMT detector and 1 sec scan rate to accumulate the spectra.

Time-correlated single photon counting (TCSPC): TCSPC lifetimes were carried out using FLS1000 Photoluminescence Spectrometer (Edinburgh instruments), equipped with a 510 nm variable pulse length diode laser (VPL-510) as the excitation source and a visible PMT-900 detector. All data analysis carried out using Floracle® software. The accuracy of the fit of the decays was judged by chi-squared (χ^2) and sum of residuals was always $\chi^2 < 1.1$. The fluorescence decay time (τ) was obtained from the slope of the decay curve. All samples were measured in a 1 cm x 1 cm quartz cuvette and samples were < 0.2 at 510 nm to ensure an optically dilute solution to avoid inner-filter effects.

NMR spectroscopy: ^{1}H-NMR was recorded on either a Bruker AC 400 MHz or 600 MHz spectrophotometer in $CDCl_3$ and were calibrated according to the deuterated solvent peak.

IR spectroscopy: FTIR measurements were carried out on Perkin-Elmer 2000 FTIR spectrophotometer in a liquid solution cell using spectrophotometric grade dichloromethane.**Transient absorption spectroscopy:** TA spectra recorded on the ps-ns timescale were measured in the ULTRA facility located in Rutherford Appleton Laboratories, UK. Experimental set-up is reported elsewhere.[1] ns-TA was recorded using the LP980 Edinburgh Instruments (λ_{exc} = 355 nm).

Time resolved Infrared spectroscopy: TRIR spectra were measured in the ULTRA facility located in Rutherford Appleton Laboratories, UK and all experimental parameters as per previously described with the TA measurements.

Scanning electron microscopy (SEM): SEM was performed on a Hitachi S3400n electron microscope electron microscope with a magnification x 300K, resolution 3.0 nm. The samples were mounted on carbon tabs and sputtered with gold coating before the measurement. SEM studies were performed at an acceleration voltage of 20 kV.

Size exclusion chromatography (SEC): SEC was conducted in 1,1,1,3,3,3-Hexafluoro-2-propanol (HFiP) using a PSS SECurity GPC system equipped with a PFG 7 µm 8 × 50 mm pre-column, a PSS 100 Å, 7µm 8 × 300 mm and a PSS 1000 Å, 7µm 8 × 300 mm column in series and a differential refractive index (RI) detector at a flow rate of 1.0 mL min^{-1}. The systems were calibrated against Agilent Easi-Vial linear poly(methyl methacrylate) (PMMA) standards and analysed by the software package PSS winGPC UniChrom.

1.2 Experimental Methods

Commercial reagents were used as received without further purifications. All reactions were carried out using standard Schlenk techniques. Anhydrous solvents were purchased from Sigma Aldrich®. 4-hyroxylmethylbenzaldehyde was purchased from Sigma Aldrich®.

Fluorescence quantum yield measurement: Steady-state fluorescence measurements were recorded using the FLS1000 photoluminescence spectrometer. Prior to obtaining the emission spectra, samples were diluted to ~ 0.2 abs units at λ_{exc} using the UV-vis spectrometer to inhibit inner-filter effect. The reference compound used was previously reported by Banfi et al. (3-pyridine H-BODIPY, Φ_{fl} = 0.62 in CH_2Cl_2).[2] An excitation wavelength of 490 nm was used for the samples and the standards. A slit width of 2.5 nm was used for all measurements. The compounds were measured in aerated solution at room temperature. The following formula was used to calculate the Φ_{fl}:

$$\Phi_{fl} = \Phi_{fl}^{std} \bullet \left(\frac{I_{fl}^{sample}}{I_{fl}^{std}}\right) \bullet \left(\frac{A^{std}}{A^{sample}}\right) \quad \textbf{(1)}$$

where $\boldsymbol{I_{fl}^{sample\ or\ std}}$ is the area under the curve of the emission spectra and $\boldsymbol{A^{sample\ or\ std}}$ denotes the absorbance of the sample at 490 nm prior to sample measurement.

Singlet oxygen quantum yield measurement: All singlet oxygen generation experiments were carried out by recording a near infrared emission (NIR) spectra and were recorded using an Andor InGaAs detector coupled with a Shamrock 163 Spectrograph. The excitation sources were supplied by Thorlabs and the monochromatic line used was a 530 nm diode laser. All UV spectra were recorded both before and after singlet oxygen measurements and it was ensured the optical density (OD) of the sample was below 0.3 absorbance units at the wavelength of excitation, prior to the sample and standard measurement. Standard and

sample measurements were run under the same experimental conditions using the same solvent, excitation sources and identical experimental parameters were utilised. All samples were run in aerated solvent at room temperature. Background measurements were carried out before running the samples. The singlet oxygen quantum yields were calculated using the following formula:

$$\Phi_{sample} = \frac{\Phi_{ref}(Area_{sample} \bullet Abs_{ref})}{(Area_{ref} \bullet Abs_{sample})} \quad \textbf{(2)}$$

Where Φ_{ref} is the singlet oxygen quantum yield of the standard (in the same solvent), $Area_{sample}$ and $Area_{ref}$ are the integrated area between 1200 – 1360 nm of the phosphorescence of singlet oxygen respectively, Abs_{ref} and Abs_{sample} are the absorption of both solutions at 530 nm. Rose Bengal was used as a reference, Φ_{Δ} = 0.53 in ACN.[3]

Lifetime analysis: The numerical procedure towards determining the lifetime of each decay (τ_i) and the corresponding % relativity of each lifetime component (B_i) is the Marquardt-Levenberg algorithm, ultimately to produce a "goodness of fit", χ^2 value:

$$\chi^2 = \sum_k w_k^2 \, (F_k - S_k)^2 \quad \textbf{(3)}$$

where χ^2 for each lifetime fit was ~ 1.2.

Time resolved kinetic analysis: For TA and TRIR, either decay or growth of bands were

fitted using either mono **(4)** or biexponential fitting **(4)** using OriginLab® software and R^2 value > 0.99 was obtained for every lifetime reported, using the followed formula for exponential fitting:

$$y = y_0 + Ae^{-x/\tau_1} \quad \textbf{(4)}$$

$$y = y_0 + A_1e^{-x/\tau_1} + A_2e^{-x/\tau_2} \quad \textbf{(5)}$$

2 Synthesis

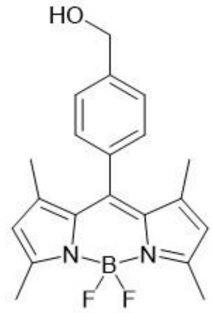

8-(4-Hyroxymethylphenyl)-4,4-difluoro-1,3,5,7-tetramethyl-4-bora-3a,4a-diaza-*s*-indacene (BODIPY-2H, monomer): The synthesis of BODIPY monomers were carried out as previously reported with some minor modifications.[4] To an oven dried flask fitted with magnetic stir-bar, 25 mL CH_2Cl_2 was added and allowed to purge with N_2 for 15 min. To the degassed solvent, aldehyde (2.5 mmol) and 2,4-dimethylpyrrole (0.51 mL, 5.0 mmol) were added. A few drops of TFA was added and the solution was stirred overnight at room temperature, in the absence of light. DDQ (2.48 mmol) was added and the solution was continuously stirred for further 4 h. After this time, boron trifluoride diethyl etherate (18.4 mmol) was added, following in quick succession by TEA (41 mmol). The reaction was stirred overnight at room temperature under a continuous flow of nitrogen. Then the reaction was stopped, and the organic layer was collected after washing three times with saturated sodium bicarbonate solution. The organic layer was collected and dried over magnesium sulphate to yield a dark purple crude product. Purification was carried out using column chromatography with on silica (hexane: ethyl acetate 70:30) to yield a red solid. ^{1}H NMR (600 MHz, $CDCl_3$): δ 1.37 (s, 6H), 1.80 (broad s, 1H), 2.55 (s, 6H), 4.81 (s, 2H), 5.97 (s, 2H), 7.27 (d, 2H, J = 8.2 Hz), 7.49 (d, 2H, J = 8.2 Hz). ^{13}C NMR (600 MHz, $CDCl_3$): δ 14.5, 14.6, 64.8, 121.2, 127.4, 128.2, 131.5, 134.2, 141.5, 141.9, 143.1, 155.5. NMR data is as per previously reported.[5, 6]

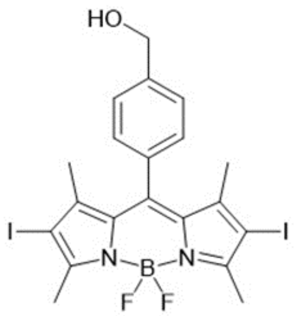

2,6-diiodo-8-(4-Hyroxymethylphenyl)-4,4-difluoro-1,3,5,7-tetramethyl-4-bora-3a,4a-diaza-*s*-indacene (BODIPY-2I, diiodo monomer): This procedure was carried out as per previous reported with some minor modifications.[2] BODIPY monomer (0.26 mmol) and Iodine (76 mg, 0.30 mmol) were added to an oven-dried round bottom flask equipped with stir-bar, dissolved in 30 mL of EtOH and purged for 30 min with nitrogen. Iodic acid (93 mg, 0.53 mmol) was dissolved in 1.5 mL deionised water and allowed to purge with nitrogen for 30 min. After that, both solutions were combined and allowed to reflux at 60 °C for 20 min. The solvent was removed using distillation, followed by dissolving the crude product in CH_2Cl_2 and washing three times with saturated sodium thiosulphate solution. The organic layer was collected, and the product was dried using rotary evaporation to yield pure product. ^{1}H NMR (600 MHz, $CDCl_3$): δ 1.32 (s, 6H), 1.52 (broad s, 1H), 2.58 (s, 6H), 4.77 (s, 2H), 7.18 (d, 2H, J = 8.4 Hz), 7.46 (d, 2H, J = 8.1 Hz). ^{13}C NMR (600 MHz, $CDCl_3$): δ 15.0, 16.1, 63.6, 84.6, 126.6, 126.9, 132.8, 140.2, 141.5, 144.3, 144.3, 155.8. MS MALDI-TOF [M]: (*m/z*) mass observed 605.9640, mass theoretical 605.9762 for $C_{20}H_{20}BF_2I_2N_2$.

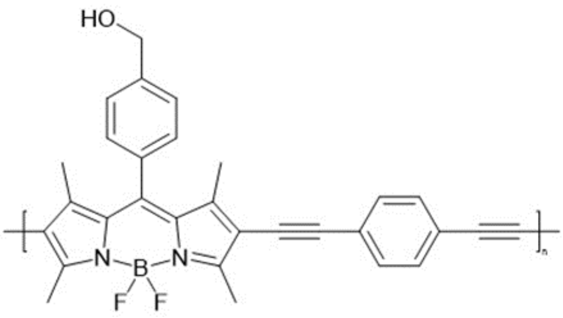

Copolymer: To an oven-dried Schlenk equipped with stir-bar, anhydrous tetrahydrofuran (THF) (15 mL) and anhydrous diisopropylamine (DiiPA) (15 mL) were added. All glass taps were greased and the Schlenk was degassed using three freeze-pump-thaw cycles. Addition of the solids to the Schlenk was then carried out - IODO-BODIPY (0.15 mmol), 1,4-diethynylbenzene (19 mg, 0.15 mmol), bis(triphenylphosphine) palladium(II) dichloride (16 mol%), copper iodide (8 mol%), triphenylphosphine (8 mol%) were added, and the freeze-pump-thaw cycle was repeated to ensure the reaction would occur in the absence of air. The solution was refluxed for 48 hr at 75-85 °C, until complete consumption of the IODO BODIPY was monitored by TLC. After this time, the solvents were removed by distillation. The crude polymer was dissolved in CH_2Cl_2 and washed three times with saturated sodium bicarbonate solution. The organic layer was dried over $MgSO_4$. Purification was carried out by washing in EtOH and collection of the precipitate to yield a pink/purple solid. This process was repeated to yield a pure copolymer free of residual catalytic system. ^{1}H NMR (600 MHz, $CDCl_3$): δ 7.69 – 7.37 (br, Ar-H), 4.83 (br, CH_2O-), 2.58 (br, CH_3 at 3, 5 position of BODIPY), 1.58 (br, OH as *meso* position of BODIPY), 1.33 (br, CH_3 at 1, 7 position of BODIPY). SEC results: M_n = 11,100, PDI = 1.1.

3 Characterisation

3.1 NMR spectra

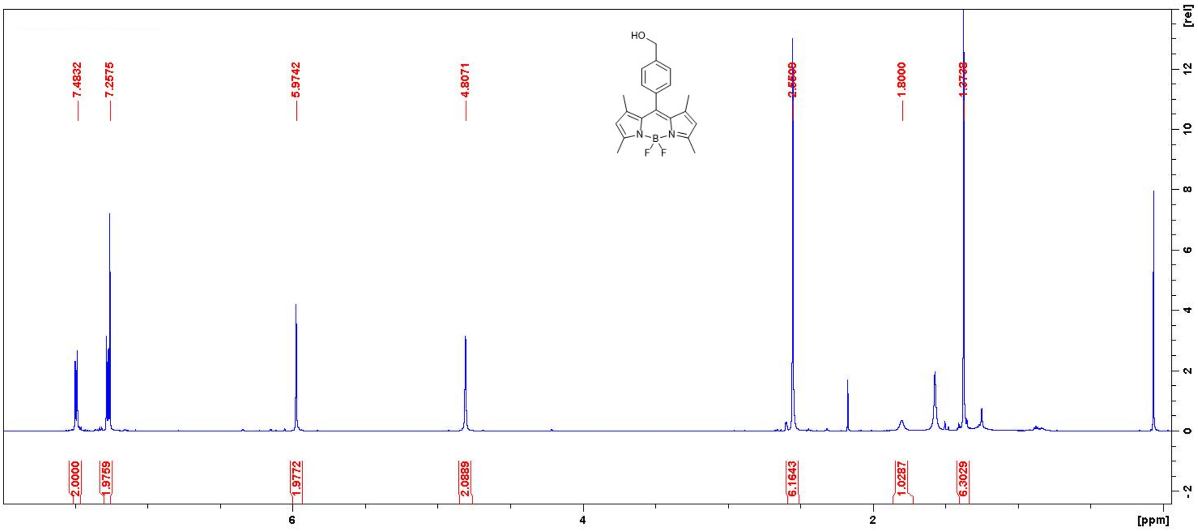

Figure S1. ^{1}H NMR (600 MHz) of BODIPY-2H in $CDCl_3$.

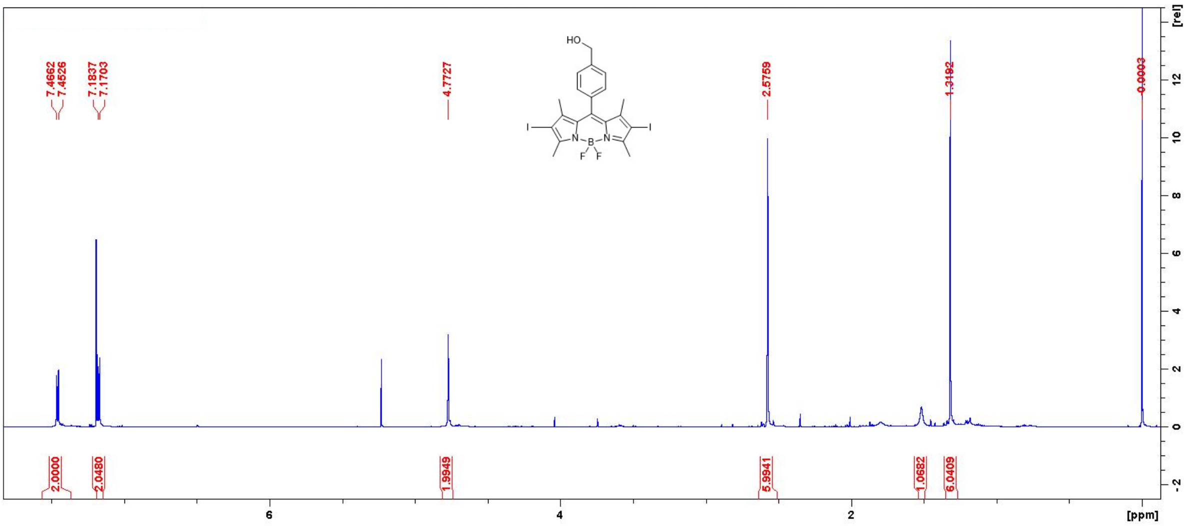

Figure S2. ^{1}H NMR (600 MHz) of BODIPY-2I in $CDCl_3$.

3.2 Mass spectroscopy

Figure: Extracted ion chromatogram (EIC) of compound.

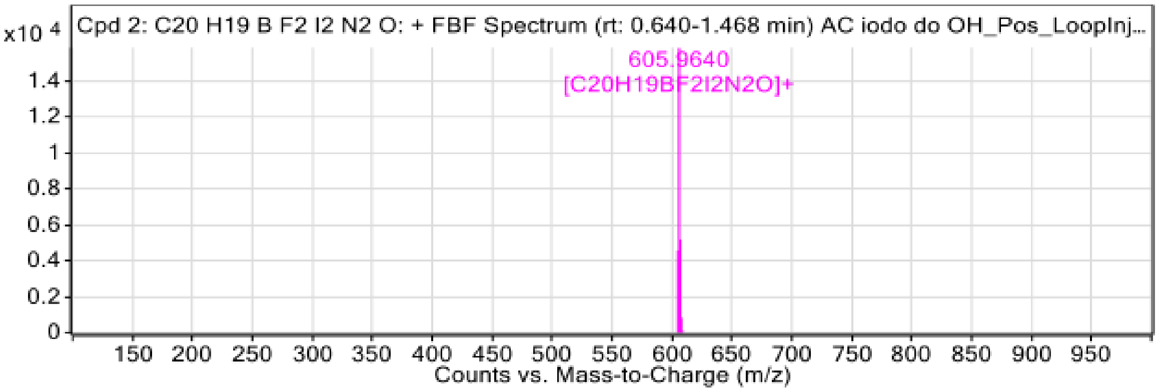

Figure: Full range view of Compound spectra and potential adducts.

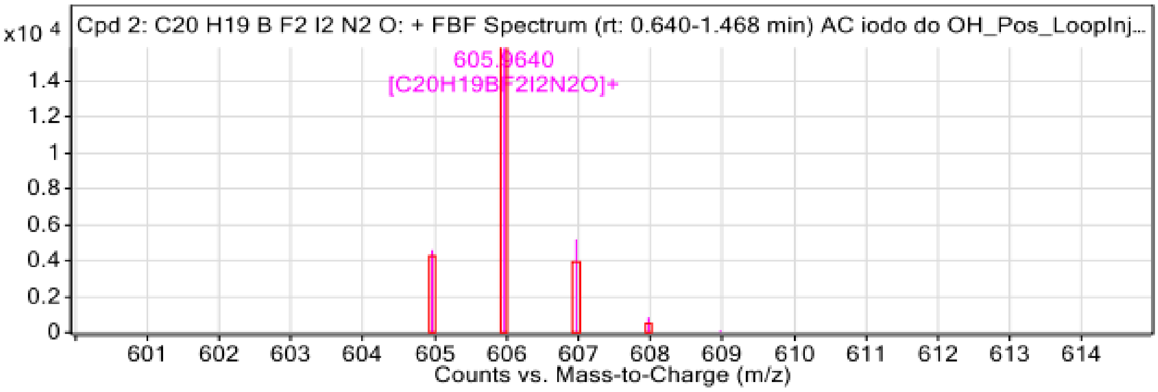

Figure S3. Mass spectra for [M^+] for BODIPY-2I, diiodo monomer.

3.3 Size Exclusion Chromatography

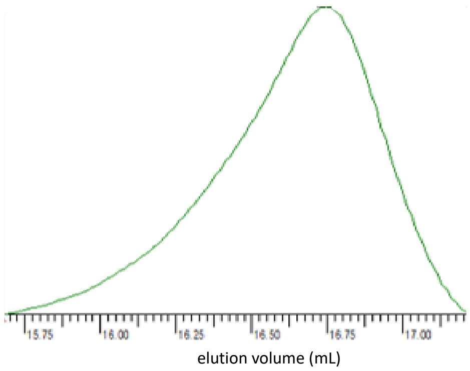

Figure S4. Size Exclusion chromatography (SEC) trace of copolymer (dRI detection, PMMA standards).

3.4 Stability of drop coated copolymer surface

The copolymer was drop coated on a cuvette surface and performed UV-Visible spectroscopy. We could observe that the OH copolymer spectra is similar in both solution and on solid surface (as shown in **Figure S5** (a)) thereby allowing to use the drop coated surface for further antimicrobial studies. Using the scanning electron microscope, we could observe the morphological appearance of the OH copolymer when drop coated on a carbon surface which is sputter coated with gold. The SEM image (**Figure S5** (b)) below show the homogeneous surface of the OH copolymer where it is unable to see and aggregation or agglomeration formation.

(a) **(b)**

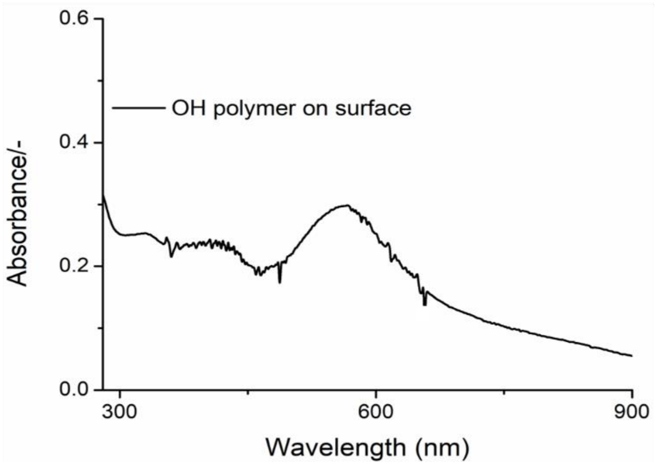

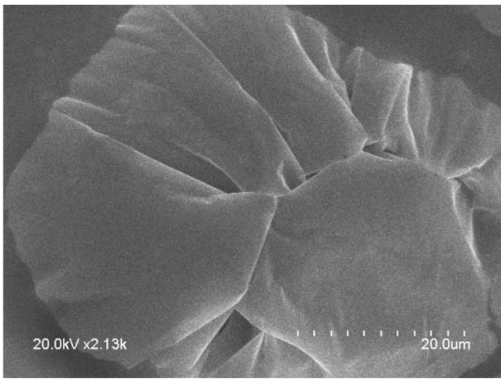

Figure S5. a) UV-visible spectra of OH copolymer drop coated onto a cuvette surface. b) SEM image representing the morphology of the OH copolymer when drop coated on a surface.

4 Absorption and emission spectra

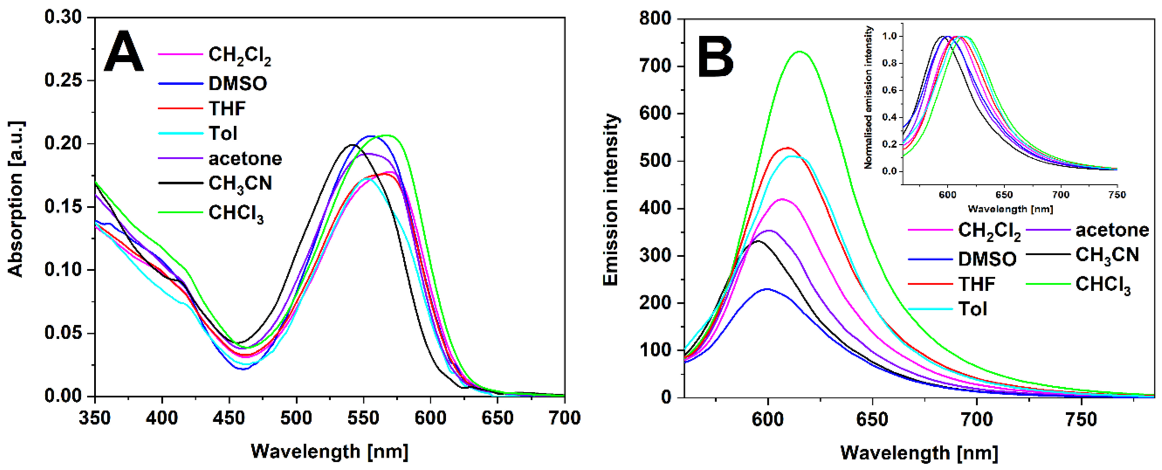

Figure S6. a) UV-visible absorption spectra of copolymer in a range of solvents; dichloromethane (solid magenta), DMSO (solid blue), THF (solid red), toluene (solid cyan), acetone (solid violet), acetonitrile (solid black), chloroform (solid green) and b) Emission spectra of copolymer in the same range of solvents, λ_{exc} = 560 nm, 15 nm slit width. Inset shows normalised emission spectra of copolymer. All spectra recorded at room temperature in aerated solution.

Table S1. Summary of photophysical properties of copolymer analysed in a range of solvents – dichloromethane, dimethyl sulfoxide, THF, toluene, acetone, acetonitrile and chloroform. The Reichardt parameter $E_T(30)$ (kcal mol^{-1}) is shown beside each solvent in brackets to indicate solvent polarity.[7] All spectra recorded at room temperature in aerated solution. 560 nm excitation wavelength was used for emission spectra with 15.0 nm slit width. All samples were isoabsorptive at the excitation wavelength. [a]calculated using the absorption maxima and emission maxima of copolymer.

	**Solvent**						
**$E_T(30)$**	**CH_2Cl_2 (40.7)**	**DMSO (45.1)**	**THF (37.4)**	**toluene (33.9)**	**acetone (42.2)**	**CH_3CN (45.6)**	**$CHCl_3$ (39.1)**
**λ_{abs} (nm)**	570	555	565	553	553	542	569
**λ_{em} (nm)**	607	599	609	611	601	595	615
**Stokes shift (cm^{-1})[a]**	1069	1324	1279	1717	1444	1643	1315

5 Lifetime measurements (TSCPC measurements)

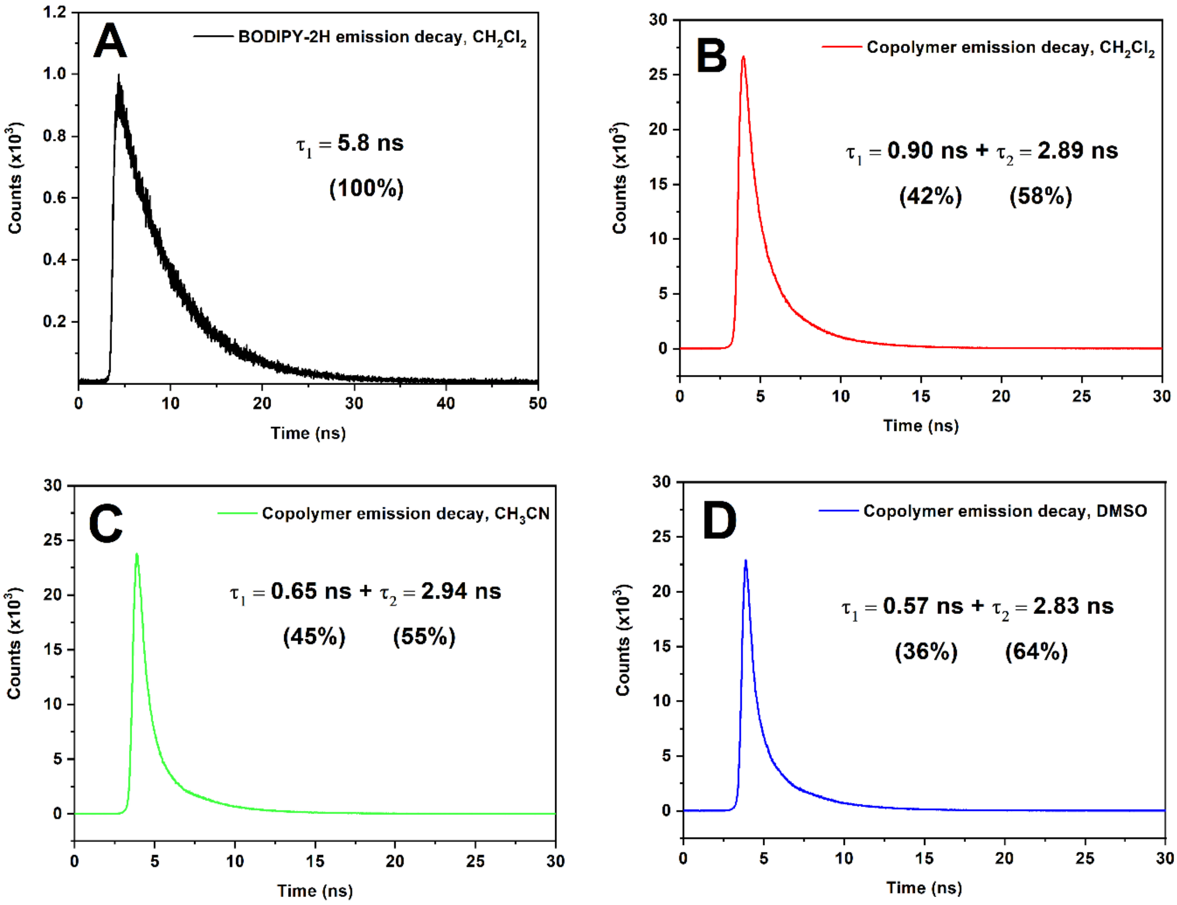

Figure S7. Emission decay profiles of a) BODIPY-2H in CH_2Cl_2, b) copolymer in CH_2Cl_2, c) copolymer in CH_3CN and d) copolymer in DMSO. The λ_{exc} = 510 nm for all samples, and a detection wavelength of 520 nm for BODIPY-2H and 610 for the copolymer. All emission decays recorded at room temperature in aerated solution.

Table S2. Fluorescence decay lifetimes (τ) including margin of error associated with each lifetime, % relativity of each component (B_i) for BODIPY-2H in CH_2Cl_2 and copolymer in CH_2Cl_2, CH_3CN and DMSO. λ_{exc} = 510 nm using FLS1000 photoluminescence spectrometer.

Compound	Solvent	τ_1 (ns)	B_1 (%)	τ_2 (ns)	B_2 (%)
**BODIPY-2H**	CH_2Cl_2	5.80+/-0.09	100	-	-
**Copolymer**	CH_2Cl_2	0.90+/-0.03	42	2.89+/-0.05	58
**Copolymer**	CH_3CN	0.65+/-0.02	45	2.94+/-0.05	55
**Copolymer**	DMSO	0.57±0.02	36	2.83±0.04	64

6 Singlet oxygen measurements

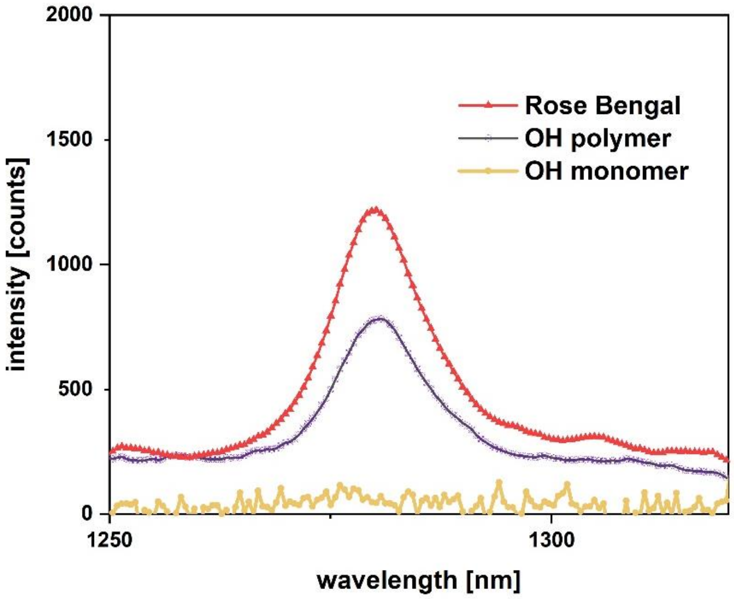

Figure S8. NIR singlet oxygen emission spectrum of Rose Bengal (red), copolymer (purple), BODIPY-2H (orange) in CH_3CN, following excitation at 525 nm.

7 Transient absorption (TA) spectroscopy

7.1 Picosecond Transient Absorption

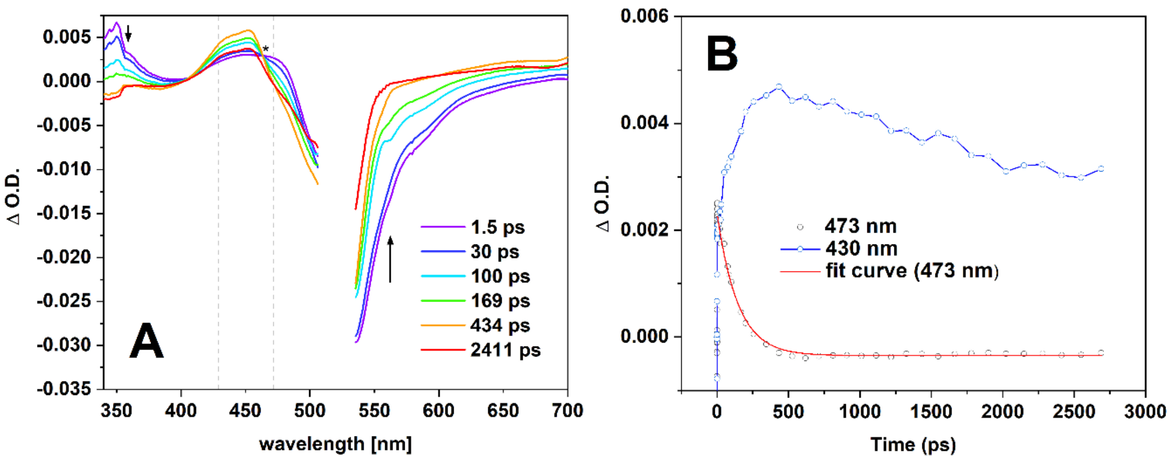

Figure S9. a) TA spectra of diiodo monomer (BODIPY-2I) in CD_3CN shown at indicated time decays in ps and b) corresponding kinetic traces at 430 nm (blue) and 473 nm (red). The red line corresponding to the kinetic trace at 473 nm shows the monoexponential fit used to obtain S_1 lifetime of halogenated species. As the signal at 473nm decays (τ = 146 ps), an additional species grows in at 430 nm (τ = 137 ps). These are assigned to the decay of the BODIPY singlet state and formation of the triplet state which persists beyond the time frame of the experimental set-up. The grey dashed lines on the TA spectra correspond to kinetic traces on the right. The asterisk indicates the isosbestic point at 463 nm. λ_{exc} = 525 nm (0.4 μJ/pulse).

Table S3. Lifetimes obtained (ps) for the species observed in the diiodo monomer at 472 and 430 nm, corresponding to decay of the singlet, and formation of the triplet state.

	τ_1 (ps)	R^2
**430 nm**	137 (± 5)	0.99
**472 nm**	146 (± 2)	0.99

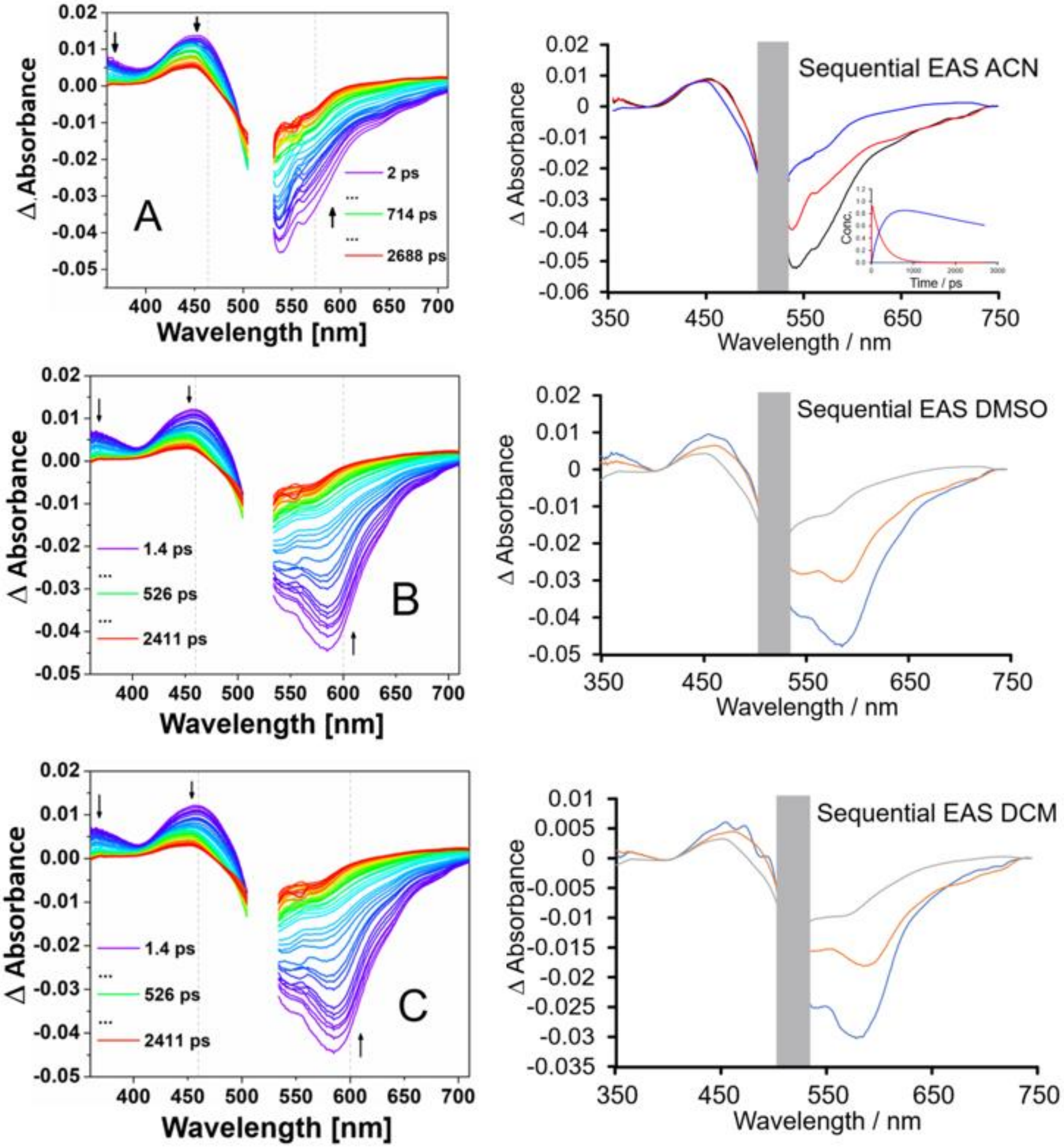

Figure S10. Transient absorption (TA) spectra of the copolymer and corresponding EAS spectra in **(A) d$_3$**-acetonitrile, **(B) d$_6$**-DMSO and **(C)** d$_2$-dichloromethane, following excitation (λ_{exc} = 525 nm (0.4 μJ/pulse) (the gap in the TA plots corresponds to the excitation wavelength which is masked by a narrow band pass filter

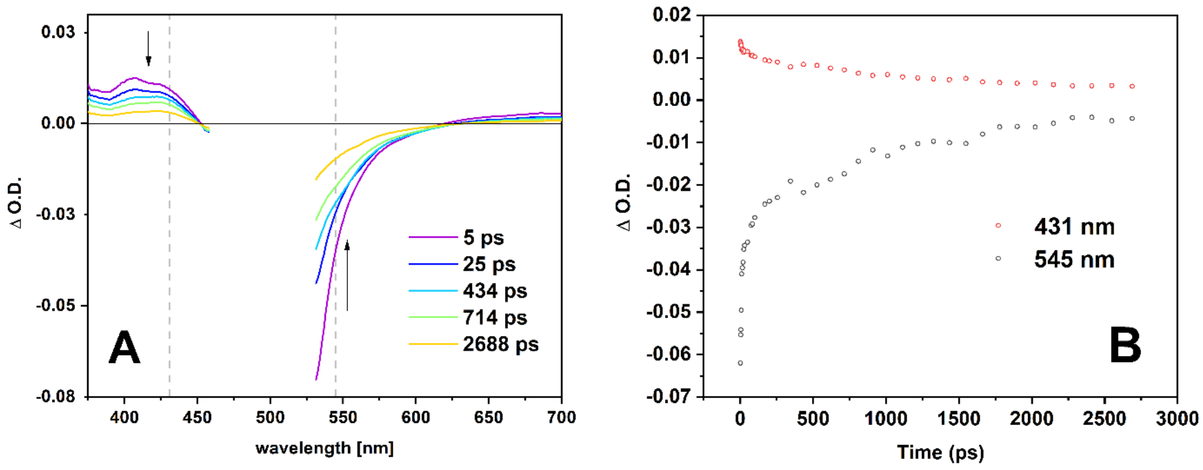

Figure S11. a) TA spectra of H-monomer (BODIPY-2H) in CH_3CN and b) corresponding kinetic traces at 431 nm (red) and 545 nm (grey). The grey dashed lines on the TA spectra correspond to kinetic traces on the right which do not decay to the baseline within the timeframe of the experiment. λ_{exc} = 525 nm (0.4 μJ/pulse).

8.2 Nanosecond Transient Absorption

Table S4. Summary of lifetimes obtained (μs) of the copolymer from a monoexponential fit of both the ESA feature (446 nm) and GSB feature (524 nm).

	τ_1 (μs)	R^2
**Acetonitrile**		
**446 nm**	32 ± 3	0.99
**524 nm**	29 ± 2	0.99

8 FTIR spectra

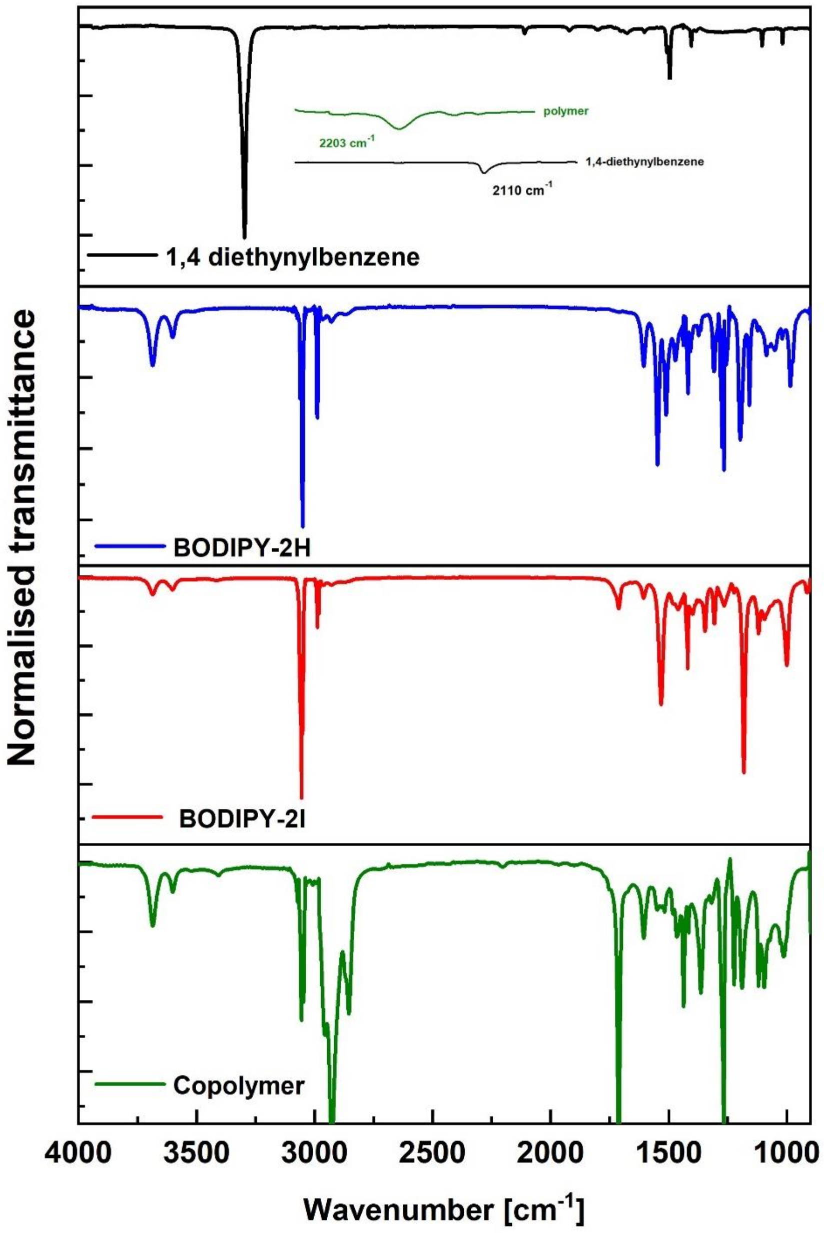

Figure S12. FTIR spectra of 1,4 diethynylbenzene (black), BODIPY-2H (blue), BODIPY-2I (red) and copolymer (green) recorded in dichloromethane solution. Inset displays triple bond region 2400 – 2000 cm^{-1}.

9 Time resolved Infrared Spectroscopy (TRIR)

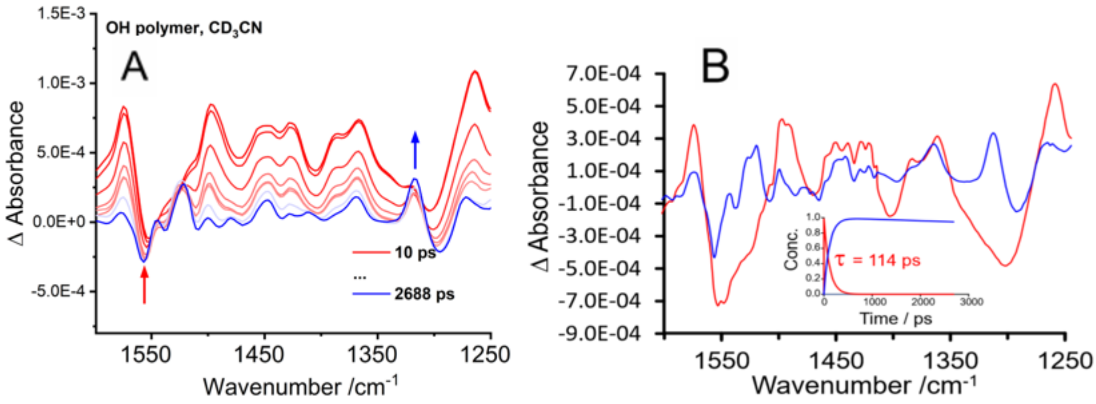

Figure S13. (A) TRIR spectra of the copolymer in CD_3CN in the spectral window of 1610 – 1290 cm^{-1} at various time delays following excitation at 525 nm, and (B) the corresponding EAS.

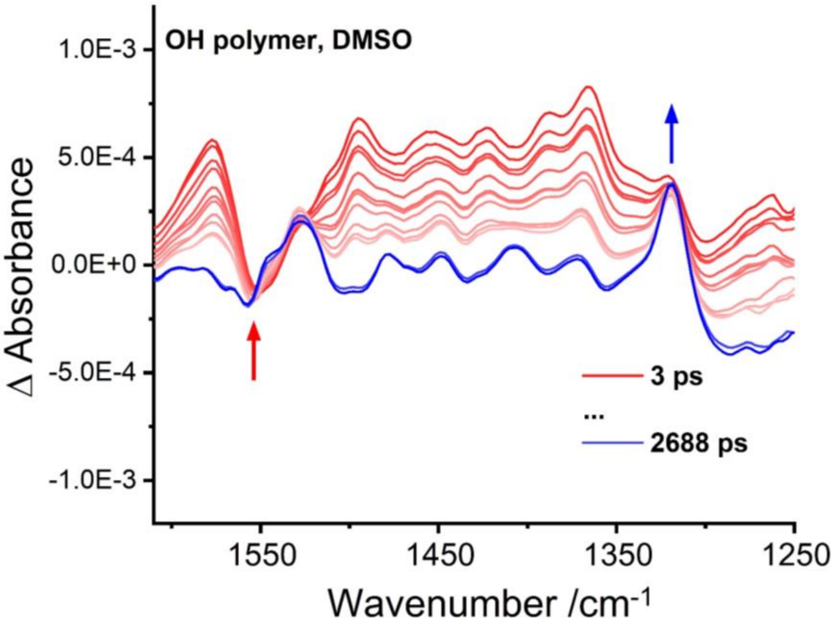

Figure S14. TRIR spectra of copolymer in DMSO in the spectral window of 1610 – 1290 cm^{-1} at various time delays following excitation at 525 nm. Arrows display spectral changes observed throughout the course of the experiment.

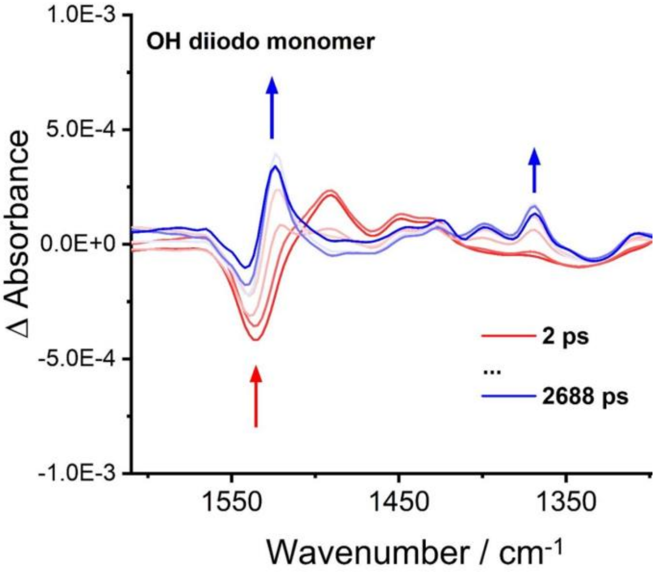

Figure S15. TRIR spectra of BODIPY-2I in CD_3CN in the spectral window of 1610 – 1290 cm^{-1} at various time delays following excitation at 525 nm. Arrows display spectral changes observed throughout the course of the experiment

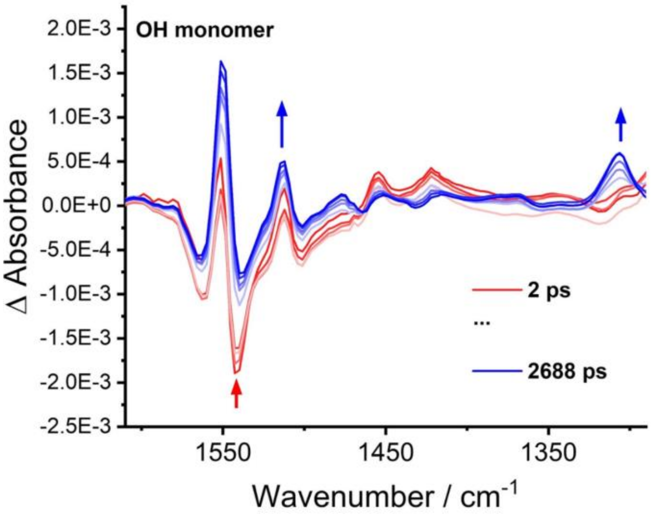

Figure S16. TRIR spectra of BODIPY-2H in CD_3CN in the spectral window of 1610 – 1290 cm^{-1} at various time delays following excitation at 525 nm. Arrows display spectral changes observed throughout the course of the experiment.

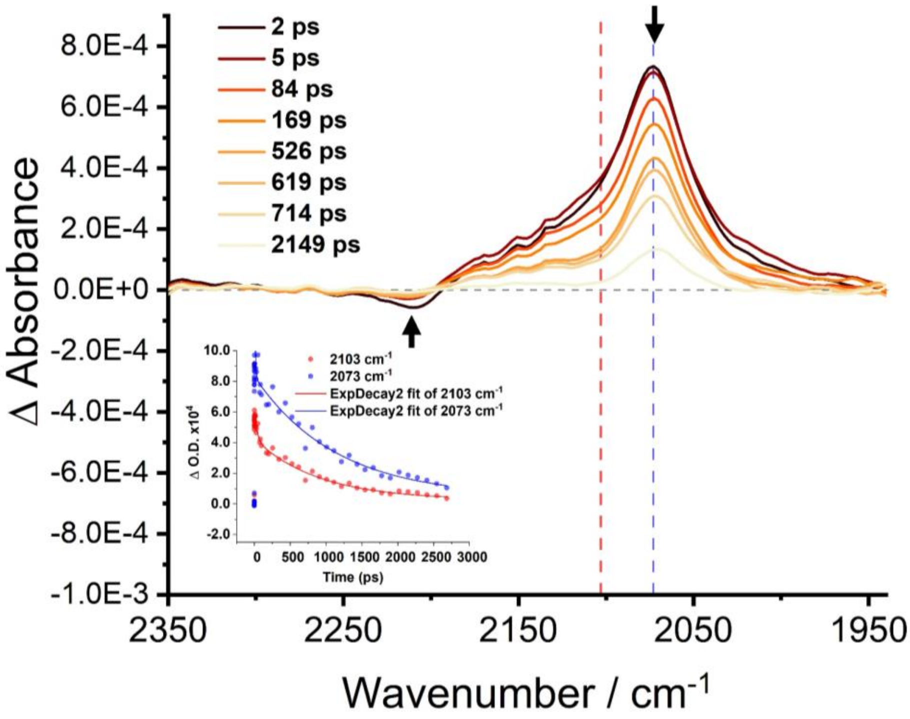

Figure S17. TRIR spectra the copolymer in $CHCl_3$ following pulsed photolysis (λ_{exc} = 525 nm) in the triple bond spectral region, recorded at various time delays, with arrows indicating the time dependent behaviour of the spectral features. Inset displays the kinetic traces at both 2103cm^{-1} and 2073cm^{-1} (corresponding to both red and blue dashed lines on spectra) and the biexponential fit (solid red and solid blue line) to obtain the decay lifetimes.

10 Antimicrobial Evaluation

The antimicrobial activity of the copolymer was assessed using four different reference bacterial strain such as *Staphylococcus aureus (S.aureus*, ATCC 25923), methicillin-resistant *S.aureus* (MRSA, ATCC 43300), *Esherichia.coli* (*E.coli*, ATCC 25922) and an extended spectrum β-lactamase (ESBL) producing *E.coli* (CL11). All the bacterial strains were grown overnight at 37°C on Mueller-Hinton (MH) agar and suspensions were prepared from isolated colonies to the density of a 0.5 McFarland standard (bioMèrieux, Ireland) and were further diluted 1/100 in phosphate buffered saline (PBS), pH7.4 (approximately 1×10^6 CFU/ml, where CFU is colony forming units). Assays were prepared in micro centrifuge tubes and contained approximately 1×10^5 CFU/mL of Gram-positive and Gram-negative bacteria with 1µg/mL (1% DMSO) and 5µg/mL (5% DMSO) of -OH polymer/monomer in phosphate buffered solution (PBS) respectively. The stability of the compound was confirmed in the solvent mixture using its absorption spectra, as shown in **Figure S17**. For irradiation, 100 µL aliquots were transferred to the wells of a 96 well tissue culture plate which was irradiated for 15 minutes using a LED lamp with wavelength ($\lambda_{exc.}$ ~ 525 nm). For non-irradiated controls, aliquots were transferred to another 96-well plate which was incubated in the dark for 15 minutes. The contents of the wells were then diluted, 1/100 with PBS and 100 µl spread onto MH agar and incubated at 37°C overnight before counting colony forming units (CFU). Control assays consisted of non-irradiated samples, addition of only DMSO and without addition of the copolymer.

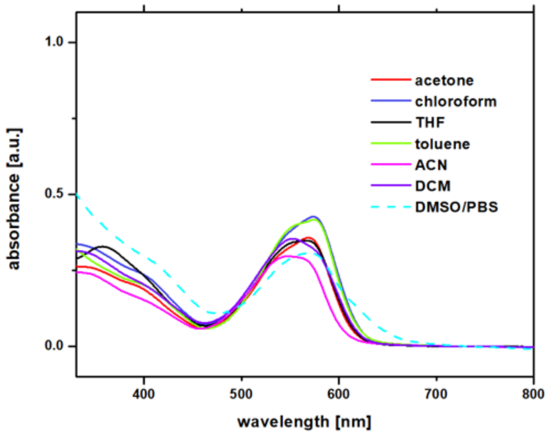

Figure S18. UV-visible absorption spectra of copolymer in a range of solvents; especially depicting the stability of the compound in a DMSO/PBS solvent mixture (cyan colour).

A comparative study on the activity of the copolymer over the monomer were performed. A similar assay as mentioned above was performed and the results are presented in **Figure S19** found below. Only on irradiation of the copolymer and monomer we can observe some bactericidal activity, where the copolymer shows an enhanced activity of > 80% killing of the gram-positive *S.aureus* and its

resistant strain. The monomer shows < 5% killing for the Gram-positive bacteria whereas > 10% killing for Gram-negative strain only with the increased concentration of the complex. As we know, Gram-negative bacteria are more resistant to antibiotics than Gram-positive bacteria, because they have an outer cell membrane. We could observe a similar trend for our complexes as it shows around 60-80 % killing for the Gram-negative *E.coli* strain was observed under higher concentration of the copolymer. The huge leap in the activity of the complex from its monomeric form to copolymer opens up new approaches towards the BODIPY in their polymeric forms.

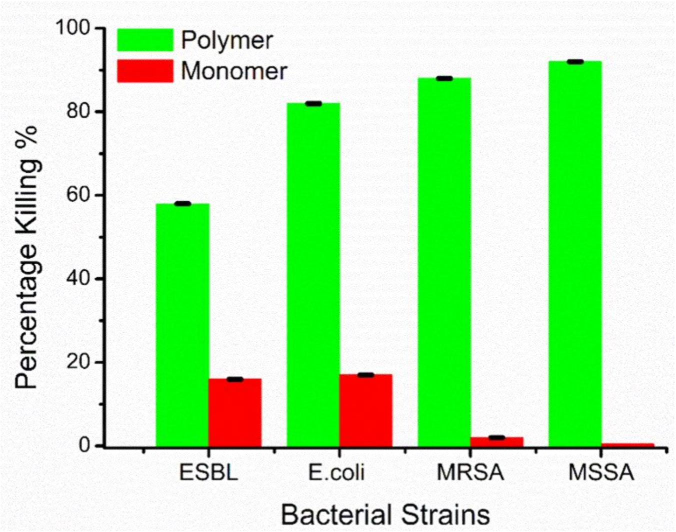

Figure S19. Comparison on the photoactivated bactericidal activity of copolymer and its BODIPY-2H, Conditions: Bacterial strains *Staphylococcus aureus* (MSSA, ATCC 25923), methicillin-resistant *S.aureus* (MRSA, ATCC 43300), *Esherichia.coli* (E.coli, ATCC 25922) and an extended spectrum β-lactamase (ESBL) producing *E.coli* (CL11), [copolymer/BODIPY-2H] for gram-positive bacteria: 1 μg/ml, [copolymer/monomer] for gram-negative bacteria: 5μg/ml, time of irradiation: 15 minutes, wavelength of light for irradiation λ ~525 nm.

11 Cytotoxic study using MTT assay

Cell lines and Culture conditions

A human keratinocyte cell line (HaCaT) obtained from the American Type Culture Collection (ATCC) was used. Cells were routinely cultured in Dulbecco's modified Eagle's medium (DMEM) supplemented with 10% (v/v) fetal bovine serum (FBS) and maintained at 37°C and 5% CO_2 atmosphere. Cells were passaged/harvested by detaching from tissue culture flasks using TrypLE ™ Express (Gibco) for 5-10 min at 37°C.

Cell Viability – MTT Assay

HaCaT cells were seeded in 96-well microtitre plates at a density of 1 x 10^5 cells/ml in 100µl culture medium (DMEM with 10% (v/v) FBS) and incubated at 37°C, 5% CO_2 for 48 h for cell attachment. Plates were washed with 100µl Phosphate Buffered Saline (PBS) and treated with novel agents (5µg to 250µg) for 2h. Control wells were treated with triton X-100 (1%) instead of test agent. Following treatment, wells were washed with 100µl PBS before adding 100µl DMEM with 10% (v/v) FBS. Plates were incubated at 37°C and 5% CO_2 for 24h. Freshly prepared MTT (3-(4,5-dimethylthiazol-2-yl)-2,5-diphenyltetrazolium bromide) (100µl of5 mg/ml in culture medium without FBS or supplements) was added to each well, followed by incubation for 4h at 37°C with 5% CO_2. The medium was discarded, and the cells were washed with PBS before adding100µl of MTT fixative solution (isopropanol) and incubating at room temperature with shaking for 4 min. Absorbance at 595 nm was measured in a Thermo Multiskan Ex plate reader.

Cytotoxicity of OH BODIPY to HaCaT cells

Measurement of OH BODIPY cytotoxicity to HaCaT cells using the MTT assay revealed a IC_{50} value of 45.2µg. This is the concentration resulting in the loss of viability of up to 50 % of HaCaT cells. This indicates some cytotoxicity to keratinocytes at concentrations close to those that resulted in > 5 log reduction in Gram-negative and >3 log reduction to Gram-positive bacterial colony forming units.

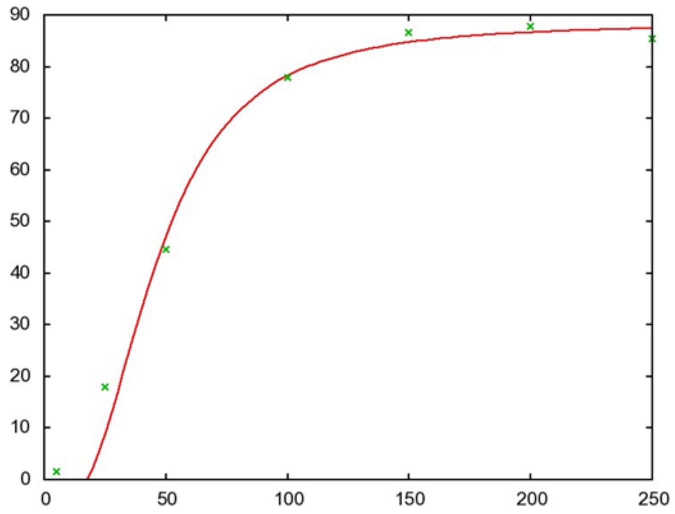

Figure S20. The half maximal inhibitory concentration (IC_{50}) curve of OH copolymer under varied concentration on the keratinocyte HaCaT cells.

12 References

1 G. M. Greetham, P. Burgos, C. Qian, I. P. Clark, P. S. Codd, R. C. Farrow, M. W. George, M. Kogimtzis, P. Matousek, A. W. Parker, M. R. Pollard, D. A. Robinson, X. Zhi-Jun and M. Towrie, *Appl. Spectrosc.*, 2010, **64**, 1311–1319.

2 S. Banfi, G. Nasini, S. Zaza and E. Caruso, *Tetrahedron*, 2013, **69**, 4845–4856.

3 N. Epelde-Elezcano, V. Martínez-Martínez, E. Peña-Cabrera, C. F. A. Gómez-Durán, I. L. Arbeloa and S. Lacombe, *RSC Adv.*, 2016, **6**, 41991–41998.

4 Y. Yue, Y. Guo, J. Xu and S. Shao, *New J. Chem.*, 2011, **35**, 61–64.

5 T. Matsumoto, Y. Urano, Y. Takahashi, Y. Mori, T. Terai and T. Nagano, *J. Org. Chem.*, 2011, **76**, 3616–3625.

6 T. Ozdemir and F. Sozmen, *RSC Adv.*, 2016, **6**, 10601–10605.

7 C. Reichardt, *Chem. Rev.*, 1994, **94**, 2319–2358.
